# Structural mechanism governing the directionality of bridge recombination

**DOI:** 10.1038/s41586-026-10903-y

**Published:** 2026-08-12

**Authors:** Masahiro Hiraizumi, Januka S. Athukoralage, Nicholas T. Perry, Eisuke Tsujimoto, Nami Shiojiri, Naoto Nagahata, Lauren Lee, Gwanggyu Sun, Matthew G. Durrant, Sita S. Chandrasekaran, Silvana Konermann, Keitaro Yamashita, Patrick D. Hsu, Hiroshi Nishimasu

**Affiliations:** 1 Department of Chemistry and Biotechnology, Graduate School of Engineering, The University of Tokyo, Tokyo, Japan; 2 Arc Institute, Palo Alto, CA USA; 3 Department of Bioengineering, University of California, Berkeley, Berkeley, CA USA; 4 San Francisco Graduate Program in Bioengineering, University of California, Berkeley, Berkeley, CA USA; 5 Department of Biochemistry, School of Medicine, Stanford University, Stanford, CA USA; 6 Structural Biology Division, Research Center for Advanced Science and Technology, The University of Tokyo, Tokyo, Japan; 7 Innovative Genomics Institute, University of California, Berkeley, Berkeley, CA USA; 8 Center for Computational Biology, University of California, Berkeley, Berkeley, CA USA; 9 Inamori Research Institute for Science, Kyoto, Japan

**Keywords:** Cryoelectron microscopy, DNA recombination, Transposition

## Abstract

Bridge recombinases from the IS110 family of transposons, such as IS621, associate with a bridge RNA (bRNA) to mediate programmable recombination between donor DNA and target DNA^1,2^. Although insertion is mediated by the recombinase–bRNA complex, it remains unknown how IS621 elements are excised from host genomes to form the circular DNA intermediates required for transposition. Here we show that bRNA is weakly expressed from IS621 loci in the *Escherichia coli* genome and that the IS621 recombinase–bRNA complex mediates excision less efficiently than insertion. Furthermore, we present the cryo-electron microscopy structures of the IS621 recombinase–bRNA complex bound to excision DNA substrates, providing mechanistic insights into the excision reaction. Similar to the previously reported donor- and target-bound insertion complex^2^, the excision complex comprises two recombinase dimers, each accommodating the target- and donor-binding loops of the bRNA. However, DNA recognition differs notably between the two complexes. Although the donor and target DNAs form a bent U-shape during insertion^2^, the excision substrates adopt linear conformations and bind across both bRNA loops, forming an X-shaped structure. This geometry reduces the efficiency of top-strand exchange and contributes to the naturally observed bias favouring insertion over excision. Despite these differences, the efficiencies of both reactions are similarly modulated by base pairing between specific dinucleotides in the bRNA, termed handshake guides, and the top strands of the DNA substrates. Overall, this study provides mechanistic insights into the complete IS110 transposition cycle and facilitates the optimal design of programmable bridge-editing applications.

## Abstract (web summary)

Cryo-electron microscopy structures show how IS621 bridge recombinase mediates DNA excision, explaining its natural preference for insertion and informing the design of programmable bridge-editing technologies.

## Main

**Fig. 1:**
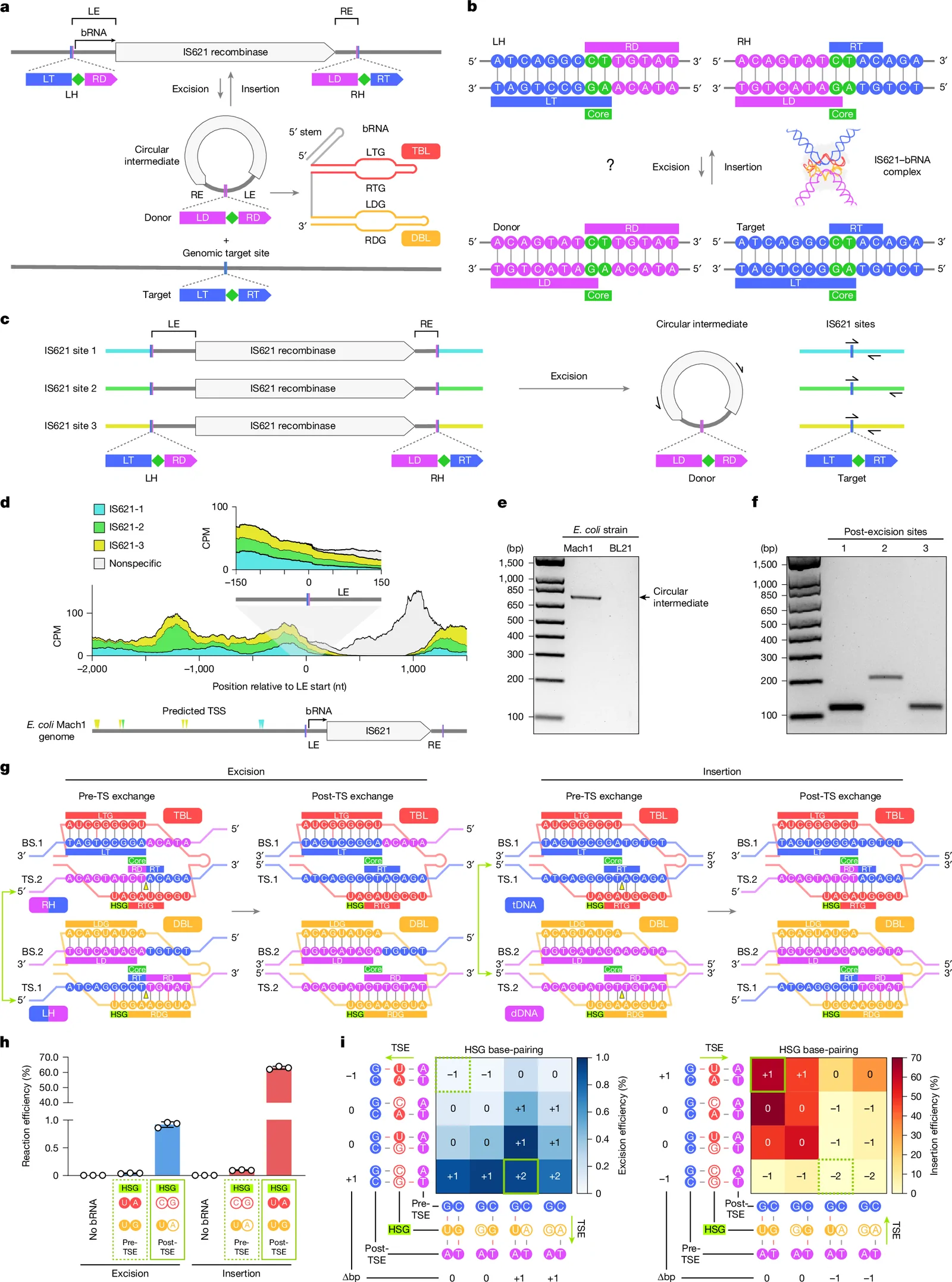
Biological and biochemical analyses of the IS621-mediated excision reaction. **a**, Schematic of the life cycle of the IS621 element. The CT core dinucleotide sequences are shown as green diamonds. **b**, Sequences of the DNA substrates for excision (left and right halves) and insertion (donor and target). **c**, Schematic of the IS621 sites in the *E. coli* Mach1 genome. Black arrows indicate PCR primers used to detect circular intermediates in **e** and post-excision sites in **f**. **d**, Transcription profiles of linear IS621 elements in the *E. coli* Mach1 genome. Mapped reads from the IS621 sites 1, 2 and 3 are overlaid. Reads that could not be assigned to specific loci due to sequence similarity were classified as nonspecific. Predicted transcription start sites (TSSs) are indicated by triangles. CPM, counts per million. **e**, Formation of circular intermediates in *E. coli* Mach1. The region spanning the LD–RD junction was amplified by PCR from *E. coli* Mach1 genomic DNA. *E. coli* BL21(DE3) was used as the control. **f**, Formation of post-excision sites in *E. coli* Mach1. The regions spanning the LT–RT junctions were amplified by PCR from *E. coli* Mach1 genomic DNA. In **e** and **f**, DNA was visualized with SYBR Gold. Data shown are representative of three technical replicates. For gel source data for **e** and **f**, see Supplementary Fig. 6. **g**, Schematic showing base pairing between the TBL/DBL in the wild-type bRNA and the DNA substrates before and after the top-strand exchange during excision and insertion. Non-canonical base pairs are indicated by red lines, and DNA cleavage sites are marked with yellow triangles. TS, top strand; BS, bottom strand. **h**,**i**, Recombination efficiencies of excision and insertion in vitro. DNA substrates were incubated with the IS621 recombinase and the engineered bRNA (RTG/RDG extensions and the G53A substitution) containing pre-top strand exchange (pre-TSE) and post-top strand exchange (post-TSE) HSG sequences (**h**) and various HSG sequences (**i**) at 37 °C for 2 h. Recombination efficiencies were quantified by qPCR. In **h**, data are shown as mean ± s.d. (*n* = 3 technical replicates). In **i**, the differences in the number of HSG–DNA base pairs before and after top-strand exchange are displayed on each cell. The efficiencies for the pre-TSE and post-TSE HSG sequences are highlighted with solid and dashed yellow-green boxes. Data are shown as mean (*n* = 3 technical replicates). TSE, top-strand exchange.

**Extended Data Fig. 1:**
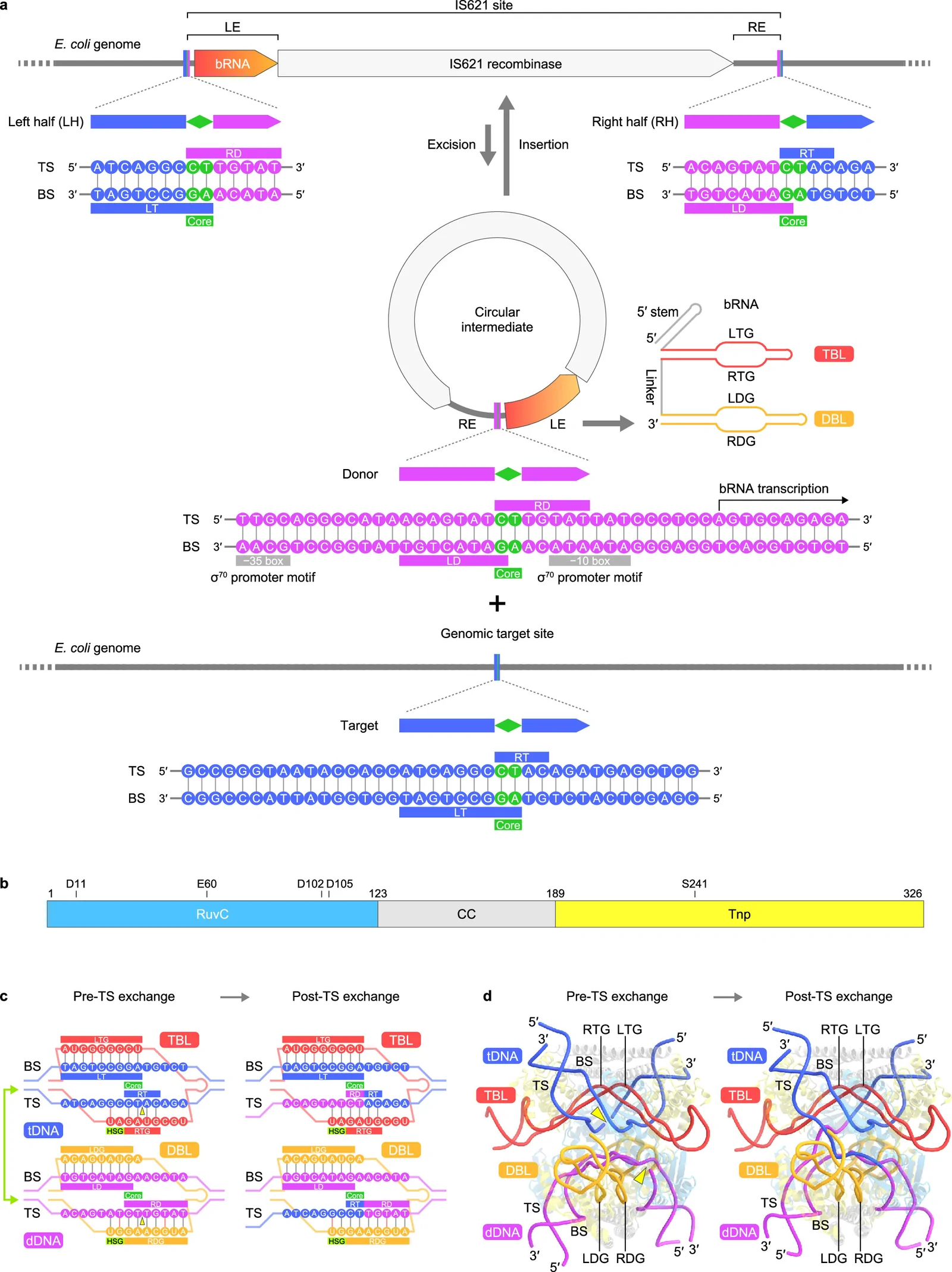
IS621 element. (**a**) Schematic of the life cycle of the IS621 element. TS, top strand; BS, bottom strand. (**b**) Domain structure of the IS621 recombinase. (**c**) Schematics of base pairing between the bRNA (TBL and DBL) and DNA substrates (tDNA and dDNA) before and after strand exchange during insertion. Non-canonical base pairing is indicated by red lines. HSG, handshake guide. (**d**) Cryo-EM structures of the IS621 insertion complex in the pre- and post-strand exchange states (PDB: 8WT7 and 8WT8)^2^. The four recombinase molecules are depicted as semi-transparent ribbon models. In (**c**) and (**d**), DNA cleavage sites are indicated by yellow triangles.

Insertion sequence (IS) elements are transposable DNA sequences found in prokaryotic genomes and are categorized into approximately 30 families, including IS110 (ref. ^3^). The transposition cycle of IS110 family elements involves two sequential recombination events: excision of the element from the host genome to form a circular double-stranded DNA intermediate and insertion of this circular form into a new target site^4–10^ (Fig. 1a,b and Extended Data Fig. 1a). Although insertion has been the focus of recent structural and functional studies^1,2^, the mechanism by which IS110 elements are excised under native conditions has remained poorly understood.

Unlike typical transposable elements^11^, IS110 elements, such as IS621 from *Escherichia coli*^6^, comprise a left end (LE), a recombinase gene and a right end (RE), with conserved CT (cytidine–thymidine) core dinucleotide sequences at both boundaries (Fig. 1a,b and Extended Data Fig. 1a). The IS110 recombinase has a distinctive domain architecture: a RuvC-like nuclease domain with a non-canonical DEDD catalytic motif (hereafter, RuvC for simplicity), a coiled-coil domain that mediates dimerization and a Tnp domain harbouring a conserved catalytic serine residue^1,6^ (Extended Data Fig. 1b). During transposition, the IS110 recombinase catalyses recombination between the circular intermediate (donor DNA (dDNA), containing left donor (LD) and right donor (RD) sequences) and the genomic target site (target DNA (tDNA), containing left target (LT) and right target (RT) sequences), generating the LT–RD and LD–RT junctions at the insertion site, referred to as the left half (LH) and right half (RH), respectively (Fig. 1b and Extended Data Fig. 1a). Excision is the reverse process: recombination of the LH and RH regenerates the circular donor and the original target site.

We recently established that the IS621 circular intermediate expresses a non-coding bridge RNA (bRNA) from a promoter reconstituted across the RE–LE junction^1^. The bRNA comprises two programmable DNA-binding loops: a target-binding loop (TBL) and a donor-binding loop (DBL), each containing guide segments that hybridize with tDNA and dDNA, respectively, enabling the IS621 recombinase to catalyse sequence-specific recombination between diverse DNA substrates^1^ (Extended Data Fig. 1c). Cryo-electron microscopy (cryo-EM) structures of the IS621 recombinase–bRNA complex bound to insertion substrates (referred to as the insertion complex) showed a tetrameric synaptic complex in which composite RuvC–Tnp active sites catalyse stepwise strand cleavage and exchange^2^ (Extended Data Fig. 1d). Furthermore, we and others demonstrated that the bridge recombinase orthologue ISCro4 can mediate programmable rearrangement of the human genome^12,13^.

Studies on the IS110 family member ISEc21 showed that a non-coding RNA derived from the upstream non-coding region co-purifies with the recombinase and is essential for circular intermediate formation, with a minimum number of target-derived flanking bases required on each side of the element for excision to proceed^14^. In a related IS110 family member, ISPpu9, multiple distinct circular intermediates are generated through recombination between different combinations of terminal sequences, in a process requiring an active recombinase and specific nucleotides at the element ends^15^. However, it remains unclear how the bridge recombinase–bRNA complex mediates excision under native conditions in bacterial cells, how the left and right element junctions are recognized, and how the bRNA is produced before circular intermediate generation, given that bRNA expression is itself driven by circularization. Here, we biochemically and structurally characterized the IS621 excision reaction, providing a resolution to this chicken-and-egg problem.

## Excision of the IS621 elements in *E. coli*

**Extended Data Fig. 2:**
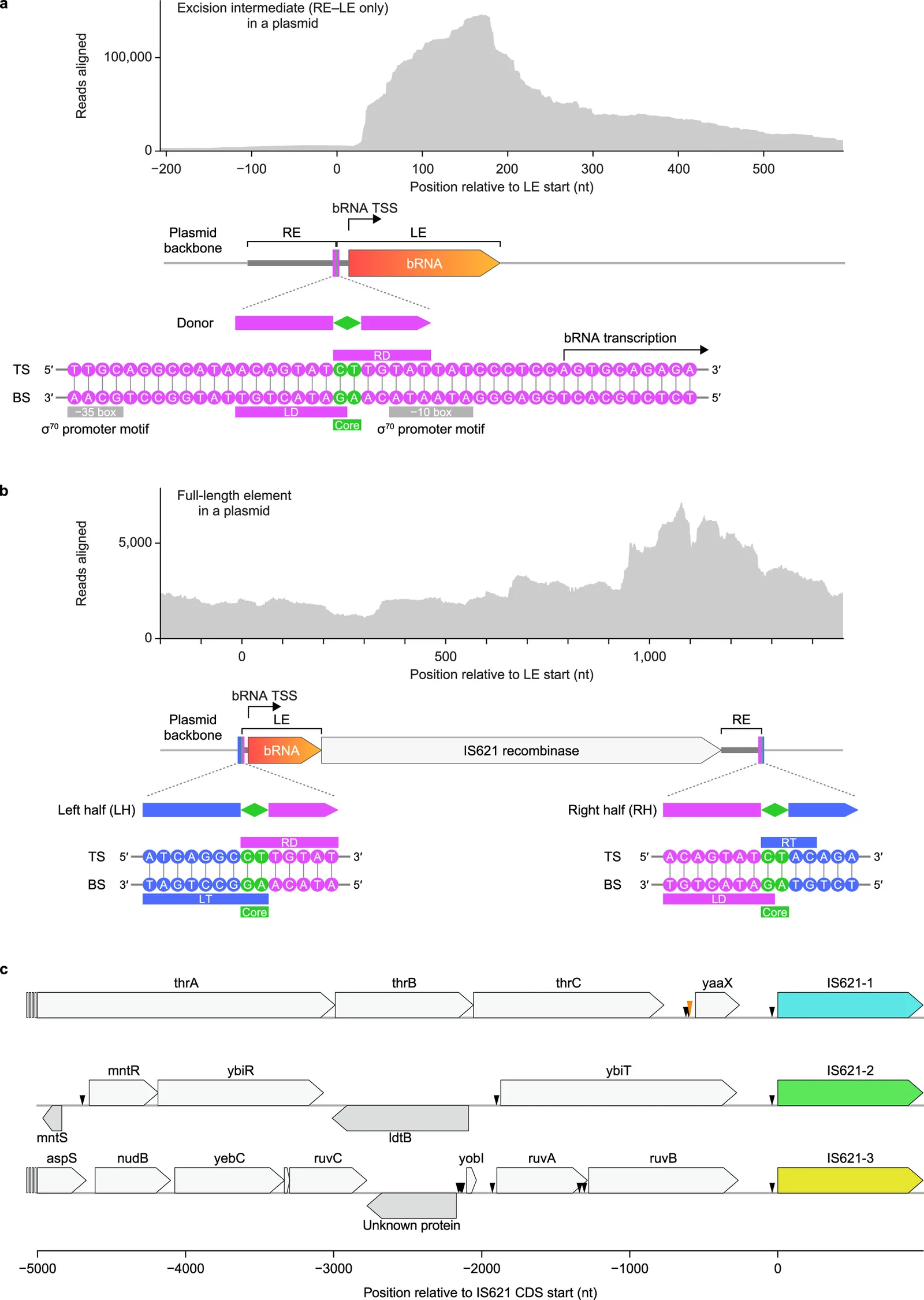
Expression profiles of the IS621 element. (**a, b**) Transcription profiles of the excision intermediate (RE–LE only) (**a**) and the full-length linear IS621 elements (LE–recombinase-coding region–RE) (**b**) encoded on a plasmid in *E. coli*. TSS, transcription start site. (**c**) Schematic of annotated coding sequences upstream of the three IS621 elements in the *E. coli* Mach1 genome. Predicted TSSs are indicated by black triangles. The orange triangle denotes the TSS upstream of IS621-1 used to design the 5′-extended bRNAs for in vitro recombination assays.

To investigate how the IS621 elements are excised by the IS621 recombinase–bRNA complex in bacterial cells, we transformed *E. coli* strain BL21(DE3), which naturally lacks IS621 elements, with plasmids encoding IS621 in one of two configurations: either mimicking the excised circular intermediate, in which the RE–LE junction is reconstituted to generate a promoter upstream of the bRNA (Extended Data Fig. 2a), or mimicking the genomic (linear) arrangement, in which the element is in its LE–recombinase–RE configuration and lacks the junction promoter (Extended Data Fig. 2b). We then profiled transcription from both constructs by RNA sequencing (RNA-seq). Consistent with our previous study^1^, we observed transcription of the bRNA from the reconstituted RE–LE junction promoter (Extended Data Fig. 2a). We also detected low-level transcription across the full-length IS621 element in the LE–recombinase–RE configuration, which lacks the junction promoter (Extended Data Fig. 2b), suggesting that both the recombinase and bRNA can be expressed at low levels even without promoter reconstitution. To assess the expression of the IS621 elements encoded in the *E. coli* genome, we sequenced the whole transcriptome of the *E. coli* strain Mach1, which contains three copies of the IS621 element (Fig. 1c and Extended Data Fig. 2c). We observed transcription originating from each genomic locus, with some transcripts extending into the region encoding the bRNA (Fig. 1d).

**Extended Data Fig. 3:**
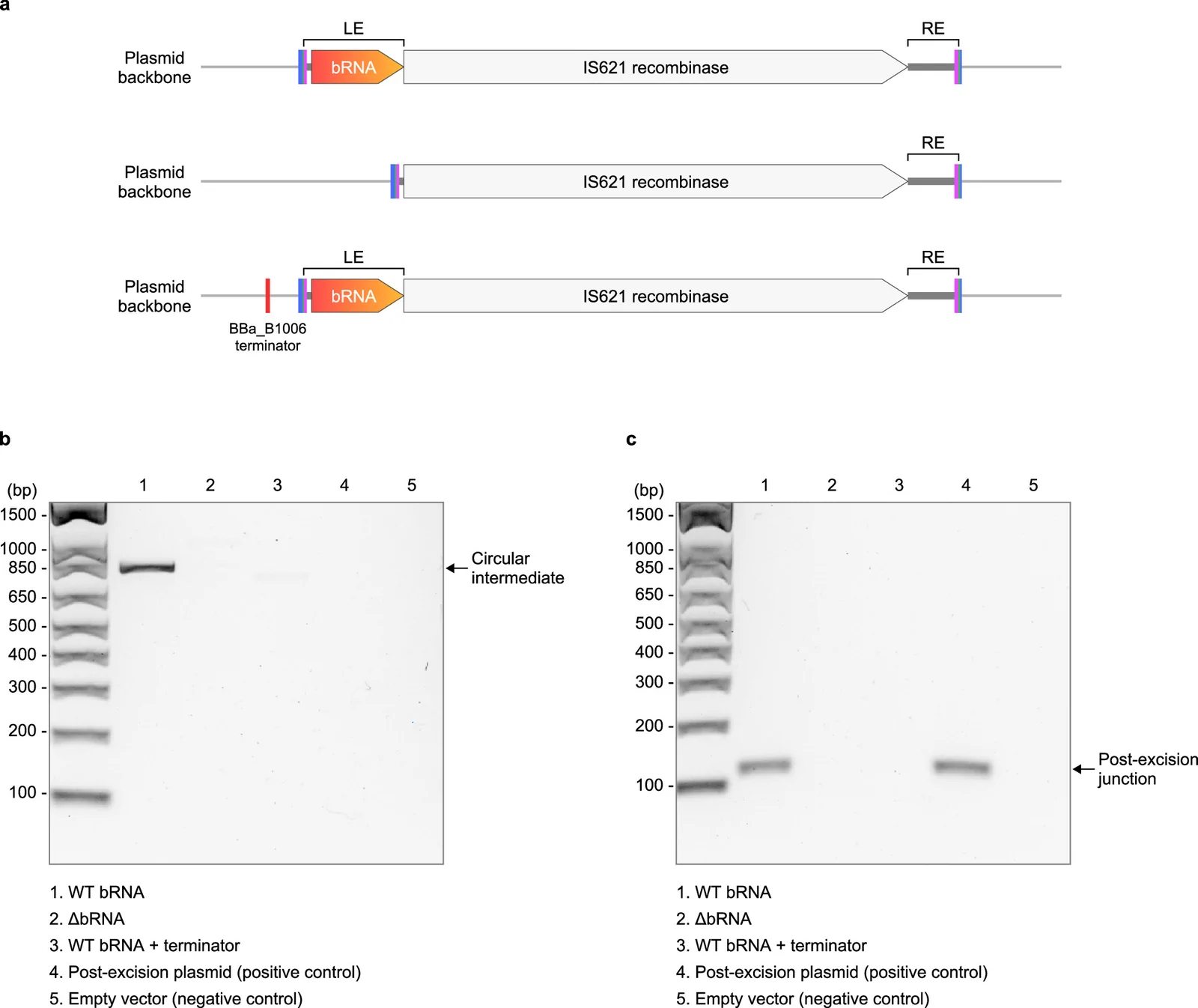
Involvement of transcription through the bRNA and the IS621 element in excision. (**a**) Schematics of plasmids encoding either the wild-type (WT) IS621 element, the element lacking the bRNA in the LE (ΔbRNA), or the element with a BBa_B1006 high-efficiency transcriptional terminator inserted upstream of the LE. (**b, c**) PCR gel images showing detection of the circular intermediate (**b**) and the post-excision (LT–RT) junction (**c**) in DNA extracted from *E. coli* BL21(DE3) cells transformed with one of the three plasmids shown in (**a**). Controls include a plasmid containing only the post-excision site (LT–RT), which should yield a signal for the post-excision junction but not for the circular intermediate, and an empty vector, which should yield neither signal. Data are representative of three technical replicates. For gel source data, see Supplementary Fig. 6.

To determine whether excision is supported by low-level transcription from the LE–recombinase–RE configuration, we performed polymerase chain reaction (PCR) targeting both the expected circular intermediate (RE–LE) and the post-excision junction in *E. coli* BL21(DE3) cells harbouring a plasmid encoding IS621 in the linear configuration (Extended Data Fig. 3a). Both products were detected from the plasmid lacking the junction promoter (Extended Data Fig. 3b,c), indicating that read-through transcription can produce sufficient amounts of the recombinase and bRNA to mediate excision. When a transcriptional terminator was inserted upstream of the LE on the plasmid (Extended Data Fig. 3a), neither the expected circular intermediate nor the post-excision junction was detected by PCR (Extended Data Fig. 3b,c), indicating that transcription through the element is required for excision. Deletion of the bRNA-encoding region from the LE yielded similar results (Extended Data Fig. 3b,c), confirming that the bRNA itself, rather than the process of transcription, is essential for excision.

We next asked whether excision also occurs from native IS621 loci in the *E. coli* genome. PCR analysis of total DNA from *E. coli* Mach1 detected the expected circular intermediate (RE–LE), whereas no product was observed in *E. coli* BL21(DE3), which lacks IS621 elements (Fig. 1c,e). Furthermore, PCR analysis of total DNA from *E. coli* Mach1 detected post-excision junctions at the expected genomic sites of all three elements (Fig. 1c,f), suggesting that the circular intermediates originate from any of the three loci across the population under basal conditions. As the three IS621 copies are highly homologous, we cannot exclude the possibility that the bRNA or recombinase expressed from one locus acts in trans to facilitate excision at another. Collectively, these results indicate that the bRNA and recombinase-coding transcripts are expressed from the IS621 elements in the *E. coli* genome and mediate the excision of these elements.

## In vitro excision and insertion reactions

**Extended Data Fig. 4:**
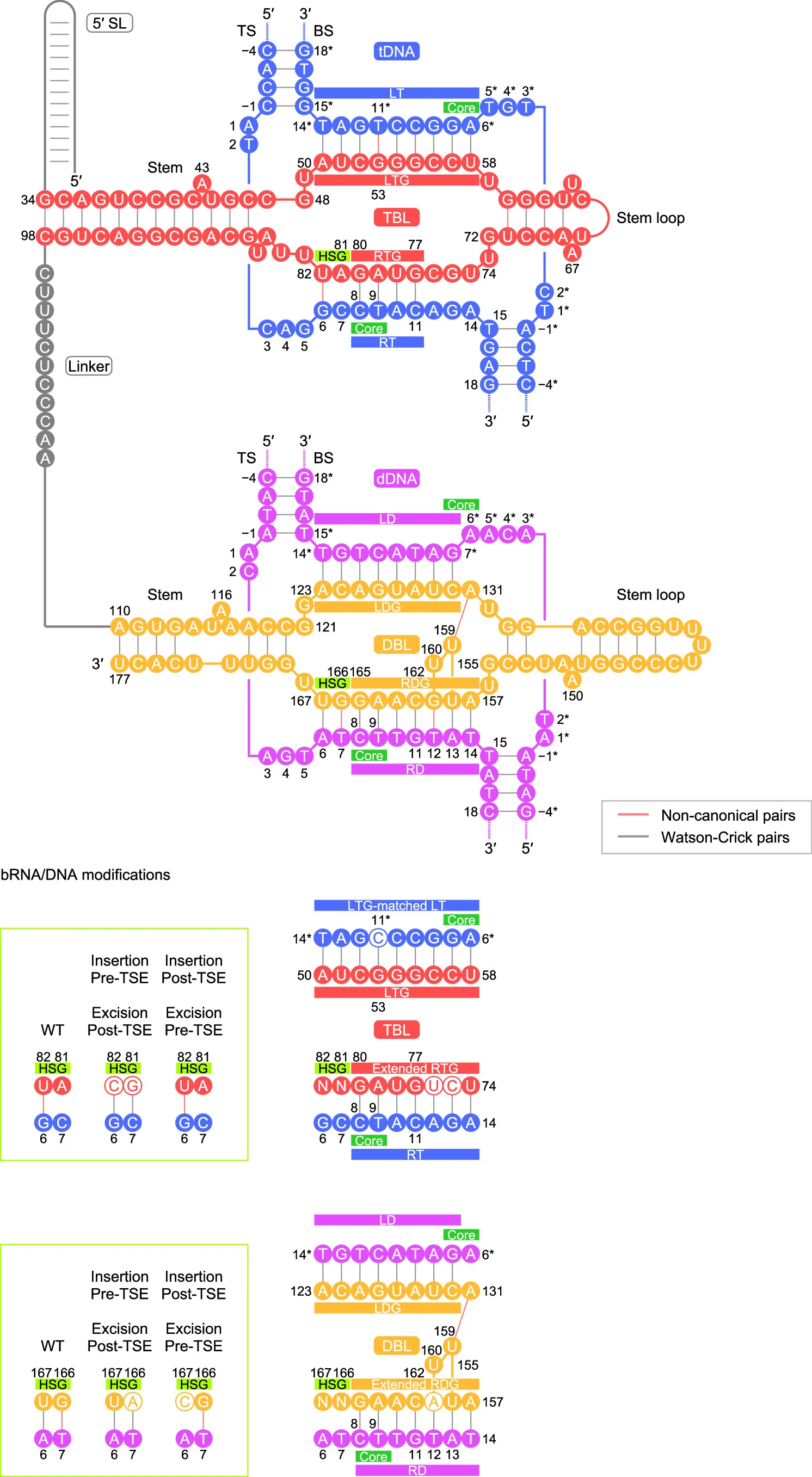
Sequences of the bRNA and DNA substrates. Sequences of the wild-type bRNA and DNA substrates for insertion (tDNA and dDNA). Nucleotide modifications are shown below, with replaced nucleotides highlighted on white backgrounds. The wild-type HSG (WT) and the pre-TSE and post-TSE HSGs are shown in yellow-green boxes. Non-canonical base pairing is indicated by red lines. HSG, handshake guide; pre-TSE, pre-top strand exchange; post-TSE, post-top strand exchange.

**Extended Data Fig. 5:**
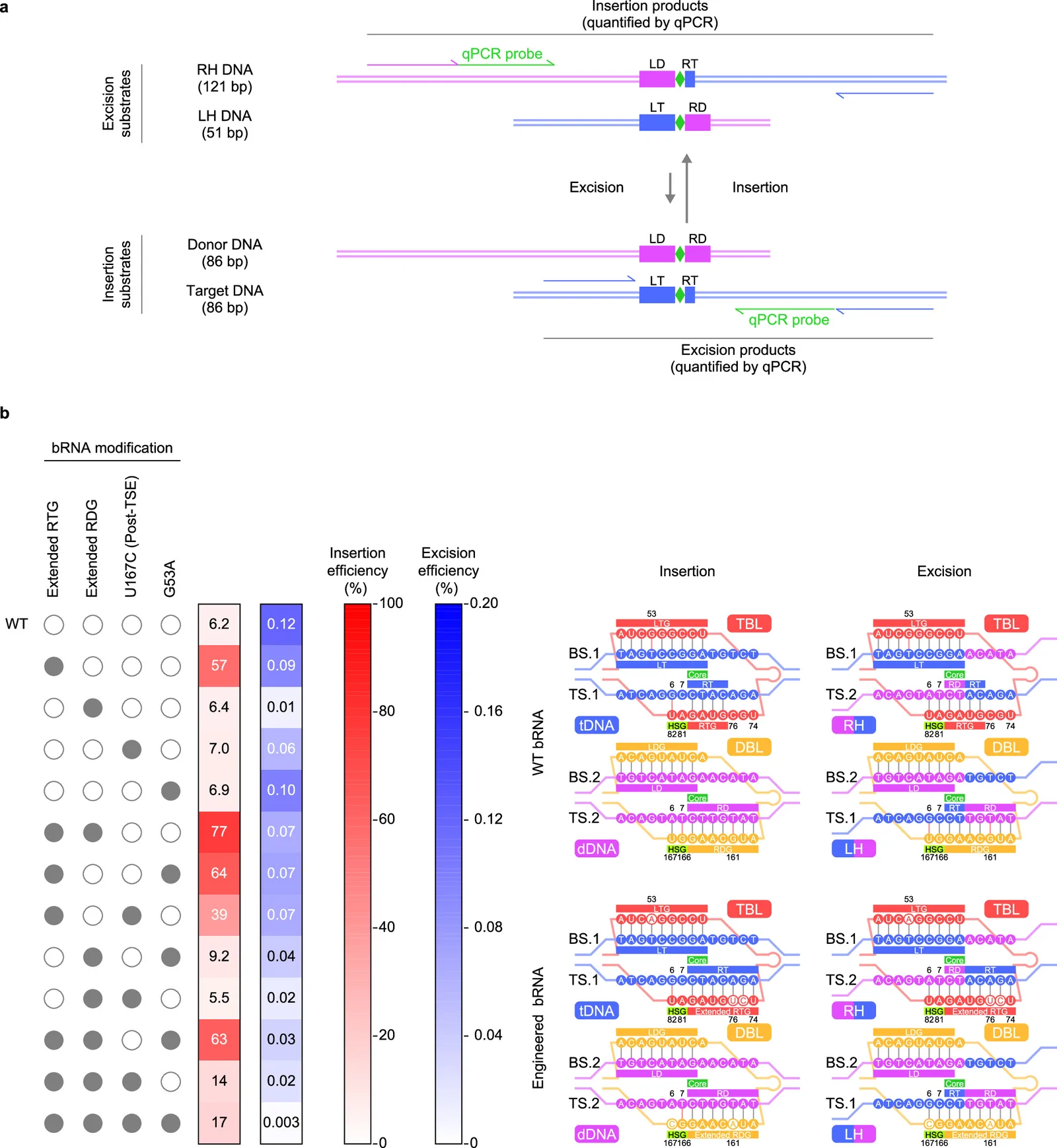
Biochemical analyses of IS621-mediated excision and insertion. (**a**) Schematic of qPCR used to quantify efficiencies of excision and insertion in vitro. (**b**) Effects of bRNA modifications on excision and insertion efficiencies. DNA substrates were incubated with the IS621 recombinase and a bRNA containing various modifications at 37 °C for 2 h. Recombination efficiencies were quantified by qPCR. Data are shown as mean (*n* = 3 technical replicates). Schematics illustrating the base pairing between the wild-type (WT) and engineered bRNA scaffolds and the DNA substrates during excision and insertion are shown on the right.

**Extended Data Fig. 6:**
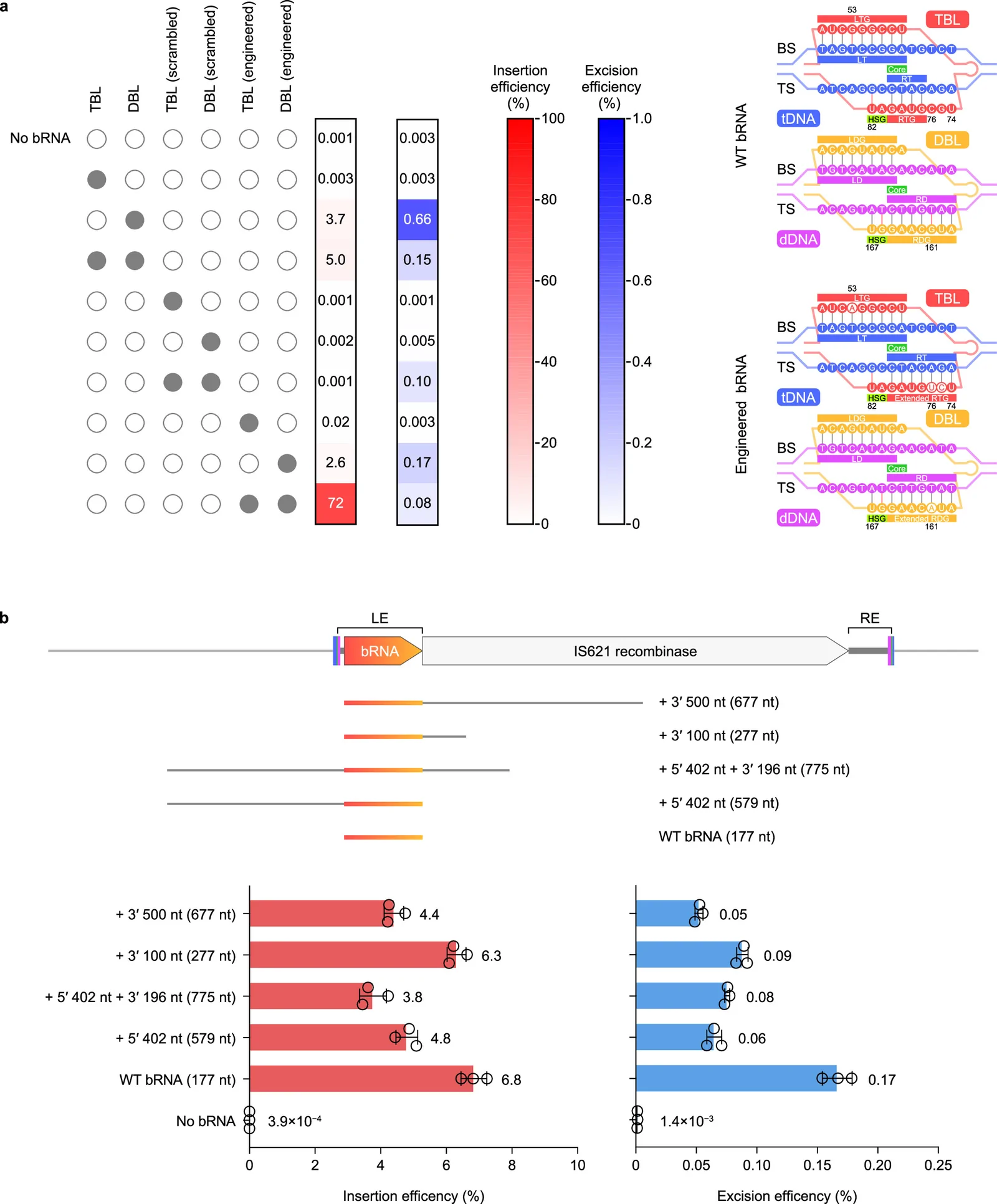
Effects of bRNA engineering on IS621-mediated excision and insertion. (**a**) In vitro recombination mediated by split TBL and DBL. DNA substrates were incubated with the IS621 recombinase and either TBL, DBL, or both (with different modifications) at 37 °C for 2 h. Recombination efficiencies were quantified by qPCR. Data are shown as mean (*n* = 3 technical replicates). Schematics illustrating the base pairing between the wild-type (WT) and engineered bRNA scaffolds and the DNA substrates during excision and insertion are shown on the right. (**b**) In vitro recombination mediated by extended bRNAs. DNA substrates were incubated with the IS621 recombinase and bRNAs extended at either the 5′ end, the 3′ end, or both, at 37 °C for 2 h. Recombination efficiencies were quantified by qPCR. Data are shown as mean ± s.d. (*n* = 3 technical replicates).

To investigate whether the IS621 recombinase–bRNA complex catalyses the excision reaction, we next measured the efficiencies of the excision and insertion reactions in vitro, using quantitative PCR (qPCR). As we previously found that IS621-mediated insertion is enhanced by extending the right-target guide (RTG; positions 74–76) and right-donor guide (RDG; position 161) segments of the bRNA^1,2^ (Extended Data Fig. 4), we assessed the impact of bRNA modifications on the efficiencies of both processes. We used the purified IS621 recombinase and the 177-nt bRNA with DNA substrates for excision (51-bp LH and 121-bp RH) or insertion (86-bp tDNA and dDNA) (Fig. 1g and Extended Data Fig. 5a). Consistent with our previous findings^1,2^, we observed substantial enhancements in insertion efficiency with the RTG extension, with further additive improvement in combination with the RDG extension (Extended Data Fig. 5b). Notably, excision was roughly 2,000-fold less efficient than insertion (0.03% compared with 63%) (Fig. 1h and Extended Data Fig. 5b), with efficient insertion requiring both the TBL and DBL (Extended Data Fig. 6a). Unexpectedly, DBL alone mediated both excision and insertion (Extended Data Fig. 6a), suggesting that DBL–DNA base pairing alone is sufficient for substrate recognition and that two DBL-containing recombinase dimers can assemble into a productive tetramer even without TBL, consistent with our previous structural observations^2^. Every bRNA modification that increased the insertion efficiency caused a corresponding decrease in the excision efficiency (Extended Data Fig. 5b).

We next explored whether extended bRNA transcripts, potentially resulting from read-through transcription originating at the promoter of an upstream gene, could support recombination in vitro. Using several transcripts (up to 775 nt) extended at the 5′ end, the 3′ end or both ends, designed based on a transcription start site mapped to an upstream coding sequence (Extended Data Fig. 2c), we found that the extended bRNAs were compatible with both excision and insertion (Extended Data Fig. 6b). When both ends were extended to mimic read-through from the upstream transcription start site into the recombinase-coding sequence, the excision and insertion efficiencies were reduced approximately two-fold relative to the minimal 177-nt bRNA (Extended Data Fig. 6b). Together, these results indicate that read-through transcription from flanking genes can produce functional bRNA transcripts that support the excision of IS621 elements from the *E. coli* genome. Overall, these biochemical data show that the IS621–bRNA complex can mediate excision, albeit with substantially lower efficiency than insertion.

**Extended Data Fig. 7:**
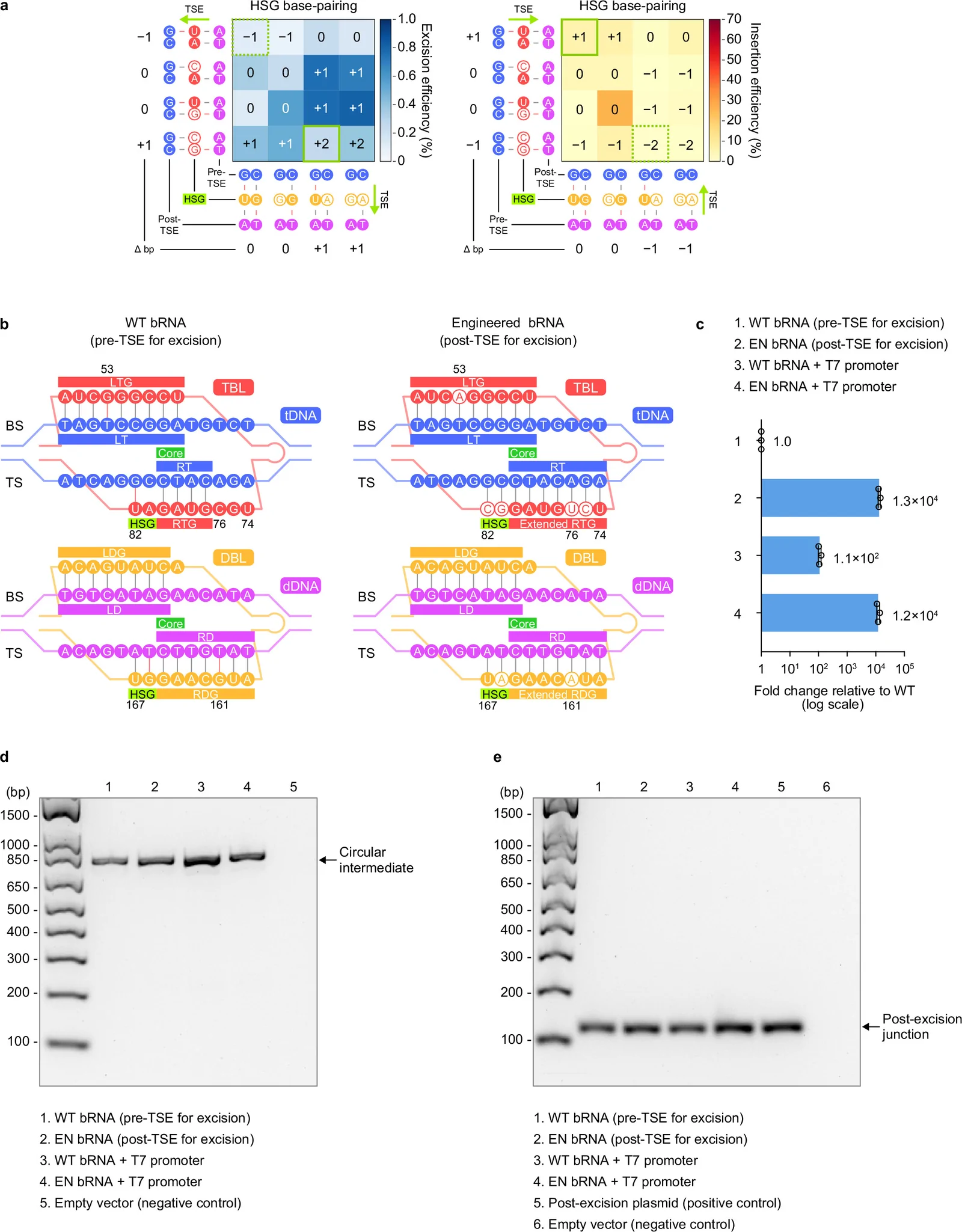
Effects of HSG–DNA base pairing on the efficiencies of excision and insertion. (**a**) Effects of HSG–DNA base pairing on the efficiencies of excision and insertion in vitro. DNA substrates were incubated with the IS621 recombinase and the wild-type bRNA containing various HSG sequences at 37 °C for 2 h. Recombination efficiencies were quantified by qPCR. The differences in the number of HSG–DNA base pairs before and after top-strand exchange are displayed on each cell. The efficiencies for the pre-TSE and post-TSE HSG sequences are highlighted with solid and dashed yellow-green boxes. Data are shown as mean (*n* = 3 technical replicates). TSE, top-strand exchange. (**b**) Schematics of the wild-type bRNA (pre-TSE for excision) and the engineered bRNA (post-TSE for excision). (**c**) Relative contributions of bRNA engineering and expression to excision efficiency. *E. coli* BL21(DE3) cells were transformed with plasmids containing the IS621 element with either the wild-type (WT) bRNA (pre-TSE for excision) or the engineered (EN) bRNA (post-TSE for excision) encoded in the LE, with or without a T7 promoter upstream of the LE. When present, T7-driven expression was induced with IPTG. Cells were grown on agar plates at 37 °C for 24 h, and excision efficiency was quantified by multiplexed TaqMan qPCR, comparing total plasmid to post-excision junction abundance from 400 ng of total plasmid DNA extracted from bulk-harvested colonies. Data are shown as mean ± s.d. (*n* = 3 biological replicates). (**d, e**) PCR gel images showing detection of the circular intermediate (**d**) and the post-excision (LT–RT) junction (**e**) in DNA extracted from *E. coli* BL21(DE3) cells transformed with plasmids encoding the wild-type (WT) bRNA (pre-TSE for excision) or the engineered (EN) bRNA (post-TSE for excision), with and without a T7 promoter driving expression across the element. Controls include a plasmid containing only the post-excision site (LT–RT), which should yield a signal for the post-excision junction, and an empty vector, which should yield neither signal. Data are representative of three technical replicates. For gel source data, see Supplementary Fig. 6.

Because the handshake guide (HSG) dinucleotides in the bRNA (positions 81–82 and 166–167) can base-pair with DNA substrates either before or after top-strand exchange^2^ (Extended Data Fig. 4), we reasoned that this pairing might modulate the reaction directionality. We investigated how the HSG–DNA base pairing affects excision and insertion, using wild-type and engineered bRNA scaffolds with four different HSGs. As reported previously^2^, insertion was facilitated by HSG–DNA base pairing after top-strand exchange, as in the natural reaction (Fig. 1h, i and Extended Data Fig. 7a). Notably, we observed an inverse trend in the relationship between the HSG sequences and the excision efficiencies (Fig. 1h,i and Extended Data Fig. 7a), suggesting that the top strands of the RD-containing LH and the RT-containing RH are recognized by the DBL RDG and TBL RTG, respectively, before top-strand exchange (Fig. 1g). Moreover, these findings indicate that in both insertion and excision, the optimal HSG configuration promotes a transition from fewer to more base pairs in the complex after top-strand exchange. We refer to the HSG configurations that maximize RNA–DNA base pairing before top-strand exchange as pre-TSE and those that maximize pairing after exchange as post-TSE. Together, these findings indicate that (1) the IS621–bRNA complex mediates both excision and insertion, (2) the HSG configuration modulates the favourability of top-strand exchange, thus determining the overall efficiency and directionality of recombination, and (3) insertion is generally more efficient than excision (for example, the efficiencies of insertion and excision were about 70% and 1%, respectively, with their post-TSE configurations) (Fig. 1h).

To determine the relative contributions of bRNA expression and sequence to the excision efficiency, we constructed plasmids encoding the IS621 element with a T7 promoter upstream of the LE in both wild-type bRNA (pre-TSE) and engineered bRNA (post-TSE) contexts. The engineered bRNA carries the post-TSE HSG configuration, RTG/RDG extensions and an LTG G53A substitution (to enable canonical A–T base pairing), which collectively promote the excision reaction (Extended Data Fig. 7b). We transformed *E. coli* BL21(DE3) with these plasmids and assessed the excision efficiency by qPCR, quantifying the post-excision junctions relative to the total plasmid. With the wild-type bRNA, the T7 promoter drove an approximately 100-fold increase in excision efficiency relative to the construct lacking a T7 promoter (Extended Data Fig. 7c), consistent with bRNA expression contributing to excision frequency. Without a T7 promoter, the engineered bRNA increased excision approximately 13,000-fold over the wild-type bRNA under the same conditions (Extended Data Fig. 7c). Adding the T7 promoter to the engineered bRNA did not further increase the excision efficiency (Extended Data Fig. 7c). Consistent with these qPCR results, both the circular intermediate and the post-excision junction were detected by PCR (Extended Data Fig. 7d,e). Together, these results indicate that the bRNA sequence, rather than its expression level, is the primary determinant of the excision efficiency, and that this barrier can be overcome by engineering the bRNA to favour strand exchange, with no additional benefit from increased transcription.

## Pre-TSE excision complex structure

**Fig. 2:**
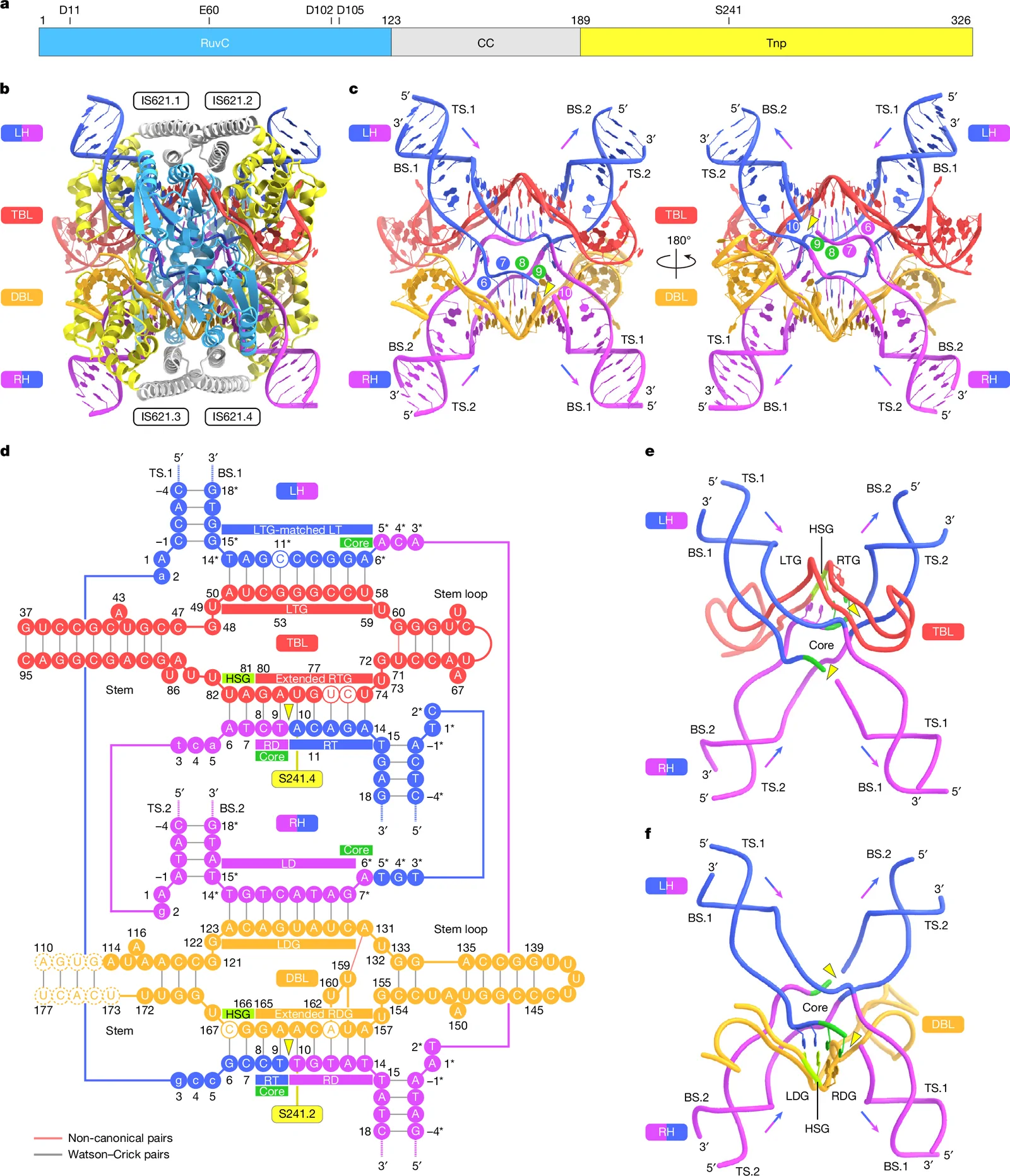
Cryo-EM structure of the IS621 excision complex in the pre-strand exchange state. **a**, Domain structure of the IS621 recombinase. CC, coiled coil. **b**,**c**, Structures of the IS621–bRNA–LH–RH complex (**b**) and the bRNA–LH–RH complex (**c**) in the pre-strand exchange state. Nucleotides surrounding the CT core (positions 6–10) are numbered, with the CT core (positions 8–9) highlighted in green. The directionalities of the LH and RH are indicated by arrows. **d**, Schematic of the bRNA–LH–RH complex. Replaced nucleotides compared with the wild-type bRNA and DNA are indicated on white backgrounds. Disordered nucleotides are indicated by dashed circles. The 5′-phosphoserine–DNA linkages are indicated by yellow lines. Non-canonical base pairing is indicated by red lines. **e**,**f**, Structures of the TBL (**e**) and DBL (**f**) bound to the LH and RH substrates. Nucleobases at positions 79–82 (TBL), 164–167 (DBL) and 6–9 (LH and RH) are shown as cartoon models to highlight RNA–DNA base pairing. In **c**–**f**, DNA cleavage sites are indicated by yellow triangles.

**Extended Data Fig. 8:**
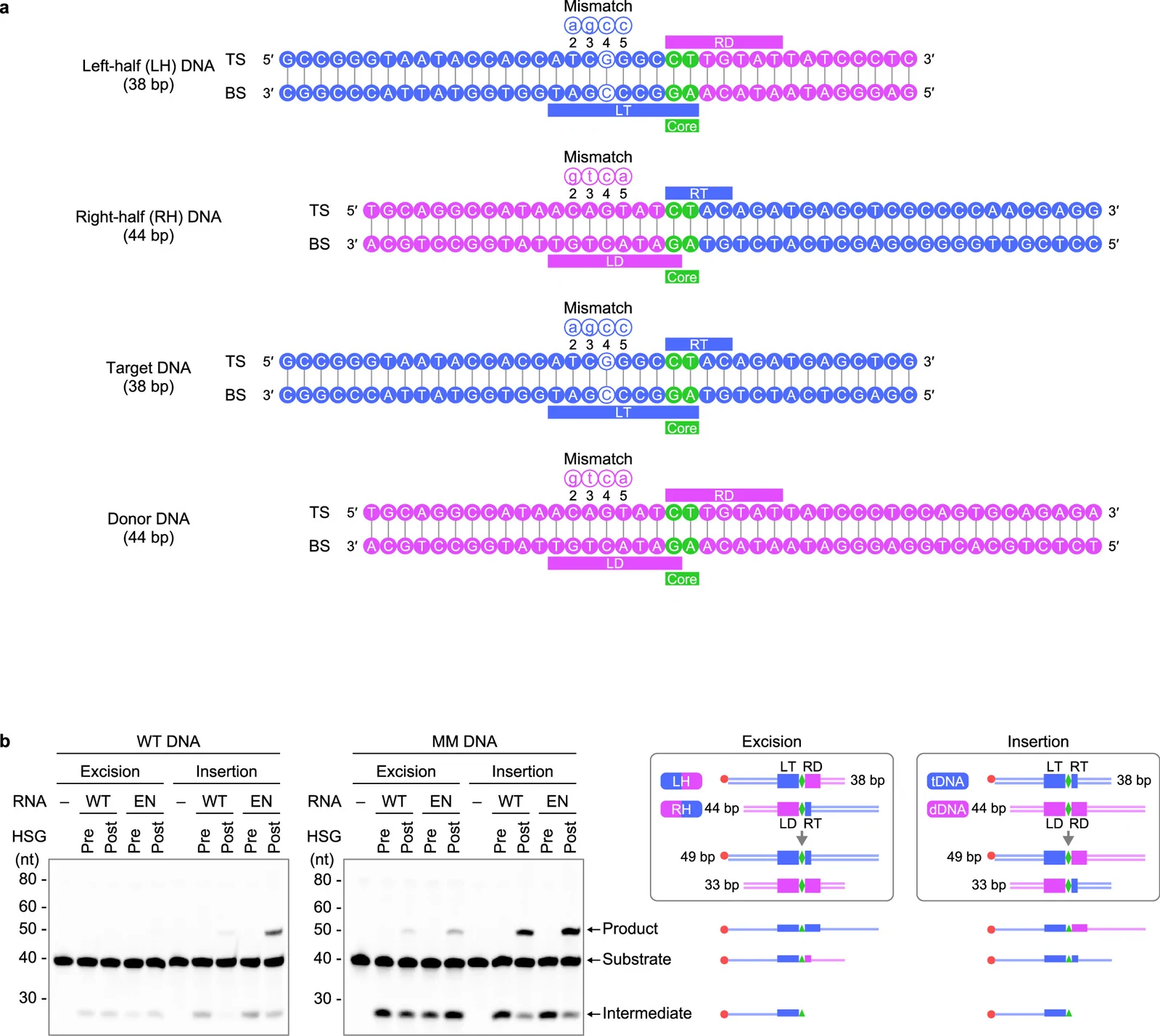
In vitro recombination assay using fluorescently-labelled DNA substrates. (**a**) Sequences of the DNA substrates for excision (LH and RH) and insertion (tDNA and dDNA) used for biochemical and structural analyses. Nucleotides substituted relative to the wild-type sequences are highlighted with white backgrounds. Mismatched nucleotides introduced to the top strands are shown as lower case letters. (**b**) In vitro recombination assay using fluorescently-labelled DNA substrates. The DNA substrates (LH/RH for excision and tDNA/dDNA for insertion) with either no mismatch (WT) or 4-nt mismatches (MM) at positions 2–5 in their top strands were incubated with the IS621 recombinase and either the wild-type (WT) bRNA or the engineered (EN) bRNA containing pre-TSE (Pre) or post-TSE (Post) at 37 °C for 1 h. The reaction products were analysed on an 18% TBE–urea denaturing gel. The LH and tDNA were labelled with Cy5 at the 5′ end of the top strand. Experiments were repeated at least three times with similar results. For gel source data, see Supplementary Fig. 6.

Although the HSG configurations modulate the relative efficiencies of both reactions, insertion is generally more efficient than excision. To explain why the IS621–bRNA complex mediates these reactions with different efficiencies, we determined the cryo-EM structure of the IS621 recombinase bound to the bRNA and its DNA substrates (referred to as the excision complex). Similar to the insertion reaction^2^, excision was enhanced by the engineered bRNA scaffold with the post-TSE HSG and DNA substrates carrying four-nucleotide mismatches at positions 2–5 in the top strand, designed to locally destabilize the DNA duplex and thereby promote base pairing with the bRNA guides (Extended Data Fig. 8a,b). Furthermore, both insertion and excision were inhibited by the bRNA with the pre-TSE HSG (Extended Data Fig. 8b). Previously, we used bRNAs containing the pre- and post-TSE HSGs to determine the structures of the insertion complexes in the pre- and post-strand exchange states, respectively^2^. Therefore, we initially reconstituted the excision complex in the pre-strand exchange state by mixing the IS621 recombinase, the engineered bRNA with the pre-TSE HSGs (A81/U82/G166/U167C), and the mismatch-containing LH and RH DNA substrates, and then determined its cryo-EM structure at 2.5-Å resolution (Fig. 2a–c, Extended Data Figs. 4 and 8a, Supplementary Fig. 1a–e and Supplementary Video 1).

**Extended Data Fig. 9:**
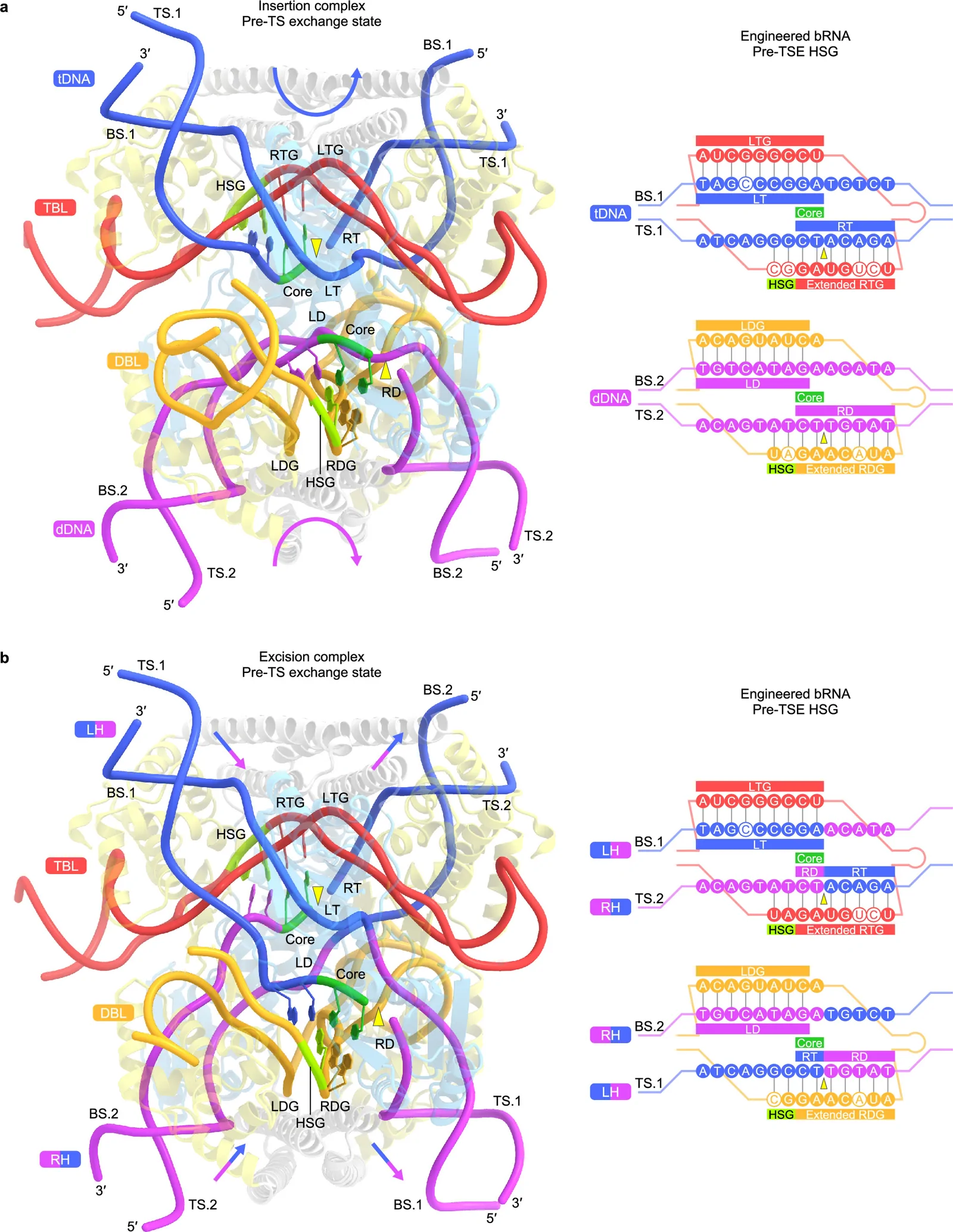
Structural comparison between the excision and insertion complexes. (**a, b**) Structures of the insertion (PDB: 8WT7) (**a**) and excision (**b**) complexes in the pre-strand exchange states. The recombinase tetramer is depicted as semi-transparent ribbon models for clarity. Nucleobases at positions 79–82 (TBL), 164–167 (DBL), and 6–9 (tDNA, dDNA, LH, and RH) are depicted as cartoon models to highlight RNA–DNA base pairing. DNA cleavage sites are indicated by yellow triangles.

The excision complex consists of four IS621 recombinase molecules (IS621.1–IS621.4), the TBL and DBL modules of the bRNA, and two DNA molecules (LH and RH) (Fig. 2b,c). As in the insertion complex^2^, the recombinase dimers (IS621.1/2 and IS621.3/4) bind the TBL and DBL, respectively, which in turn recognize the two DNA substrates through RNA–DNA base pairing (Fig. 2a–c and Supplementary Fig. 2a,b). However, the DNA substrates adopt strikingly different conformations within the insertion and excision complexes. During insertion, the tDNA and dDNA are bent into U-shaped conformations within each dimer^2^ (Extended Data Fig. 9a). During excision, the LH and RH instead adopt extended, linear conformations that cross between both dimers, producing an X-shaped arrangement (Fig. 2c and Extended Data Fig. 9b). Furthermore, the DNA substrates are recognized by the TBL and DBL differently in these two complexes. In the insertion complex, the tDNA and dDNA are recognized by the TBL and DBL within each dimer, respectively^2^ (Extended Data Fig. 9a). By contrast, in the excision complex, the LH and RH are recognized by both the TBL and DBL, resulting in each dimer interacting with both substrates simultaneously (Fig. 2c and Extended Data Fig. 9b). As in the insertion complex^2^, the catalytic S241 loops in IS621.2 and IS621.4 are ordered and interact with the D102 loops in IS621.3 and IS621.1, respectively, creating composite active sites to cleave the top strands of both DNA substrates and form 5′-phosphoserine intermediates (Supplementary Fig. 2a,b). The corresponding S241 loops in IS621.3 and IS621.1, which would mediate bottom-strand cleavage, are disordered in this structure (Supplementary Fig. 2a,b). Thus, this structure represents the top-strand cleaved, pre-strand exchange state during the excision reaction.

## Excision DNA recognition

The cryo-EM structure of the excision complex explains how the IS621–bRNA complex recognizes the excision DNA substrates (LH and RH). As in the insertion complex^2^, the TBL and DBL adopt similar pseudo-symmetric architectures and are recognized by the protein (Fig. 2d–f and Supplementary Fig. 3a). The TBL LTG and the DBL LDG base pair with the LH LT and the RH LD, respectively, whereas the TBL RTG and the DBL RDG base pair with the RH core–RT and the LH core–RD, respectively (Fig. 2d–f and Supplementary Fig. 3a). As expected, the HSG regions in the TBL (positions 81–82) and DBL (positions 166–167) base pair with the RH (A6–T7) and the LH (G6–C7), respectively, thereby preventing top-strand exchange. Unlike in the insertion complex^2^, nucleotides U49, U73, G122 and U156, which flank the four guide segments, form stacking interactions with the DNA nucleobases at positions 3 and 3*, helping the DNA strands pass across the TBL- and DBL-bound dimers (Supplementary Fig. 3a,b). Moreover, in the excision complex, U160 in the RDG adopts a different conformation compared with that in the insertion complex and stacks with A6* in the RH, contributing to DNA binding (Supplementary Fig. 3a,b).

**Extended Data Fig. 10:**
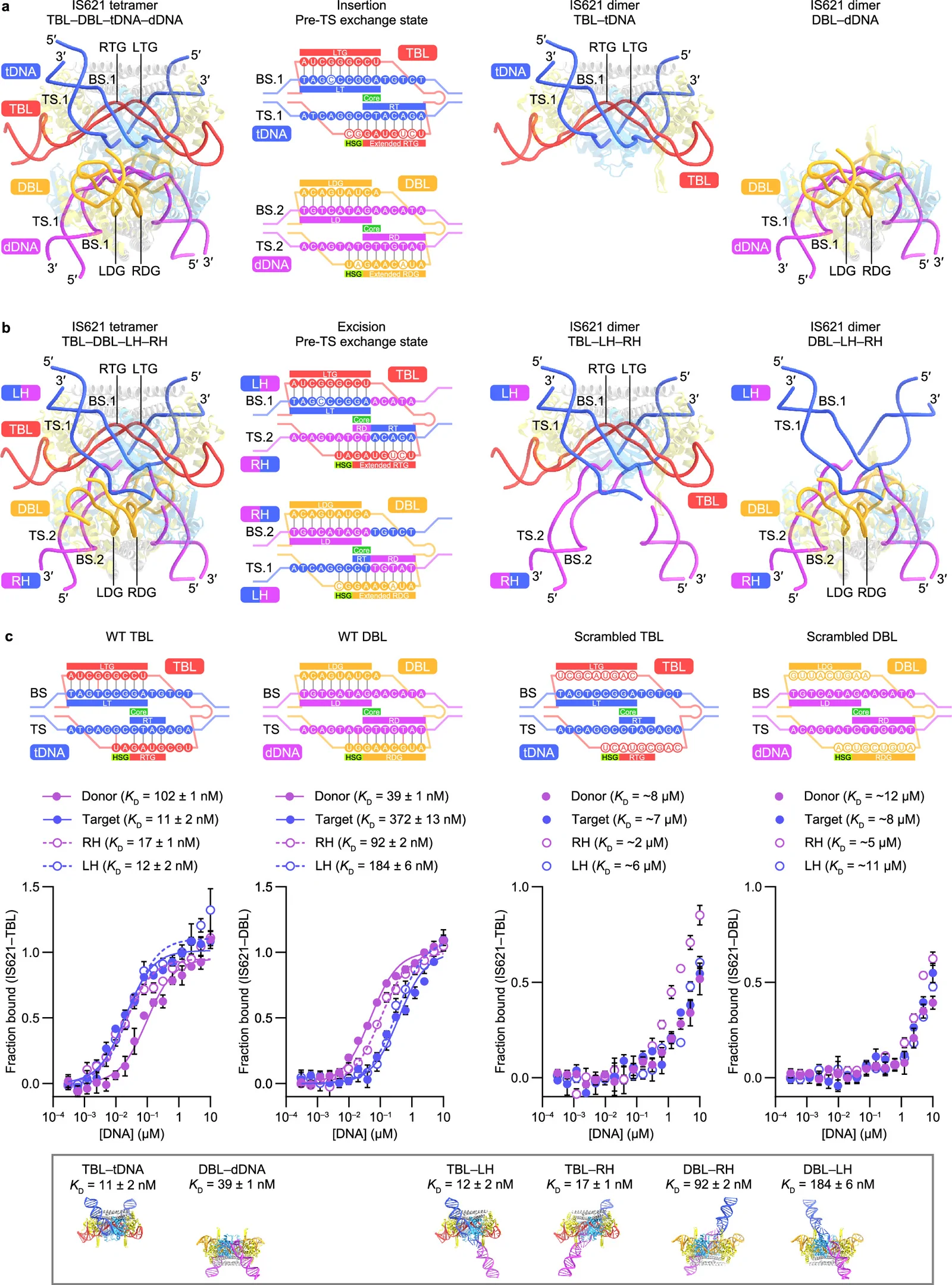
Distinct assembly mechanisms of the excision and insertion complexes. (**a, b**) Structures of the insertion (PDB: 8WT7) (**a**) and excision (**b**) complexes in the pre-strand exchange states. The DNA-bound IS621–TBL and IS621–DBL dimers, predicted based on the tetrameric complex, are shown on the right. (**c**) DNA binding to the TBL- and DBL-bound IS621 recombinase dimers. The IS621–TBL and IS621–DBL dimers were pre-assembled by mixing the IS621 recombinase with either the TBL or DBL with wild-type or scrambled guides. The IS621 dimeric complexes were incubated with different concentrations of DNA substrates (dDNA, tDNA, RH, or LH), and DNA binding was assessed by microscale thermophoresis. Structural models of the DNA-bound IS621–TBL/DBL dimeric complexes, predicted by AlphaFold3^25^, are shown below the graphs. Data are shown as mean ± s.e.m. (*n* = 3 technical replicates).

During the insertion process, the TBL- and DBL-bound recombinase dimers separately bind only the tDNA and dDNA, respectively, and then come together to form the tetrameric complex^2^ (Extended Data Fig. 10a). By contrast, the excision complex structure suggests that the TBL- and DBL-bound recombinase dimers can bind both the LH and RH substrates (Extended Data Fig. 10b). Microscale thermophoresis measurements showed that the TBL- and DBL-bound dimers each bind both LH and RH with comparable affinity, whereas they preferentially bind the tDNA and dDNA, respectively (Extended Data Fig. 10c), indicating the different assembly processes between the two complexes. Together, these data show how the IS621–bRNA complex recognizes the LH and RH sequences during the excision process.

## Excision DNA cleavage

**Fig. 3:**
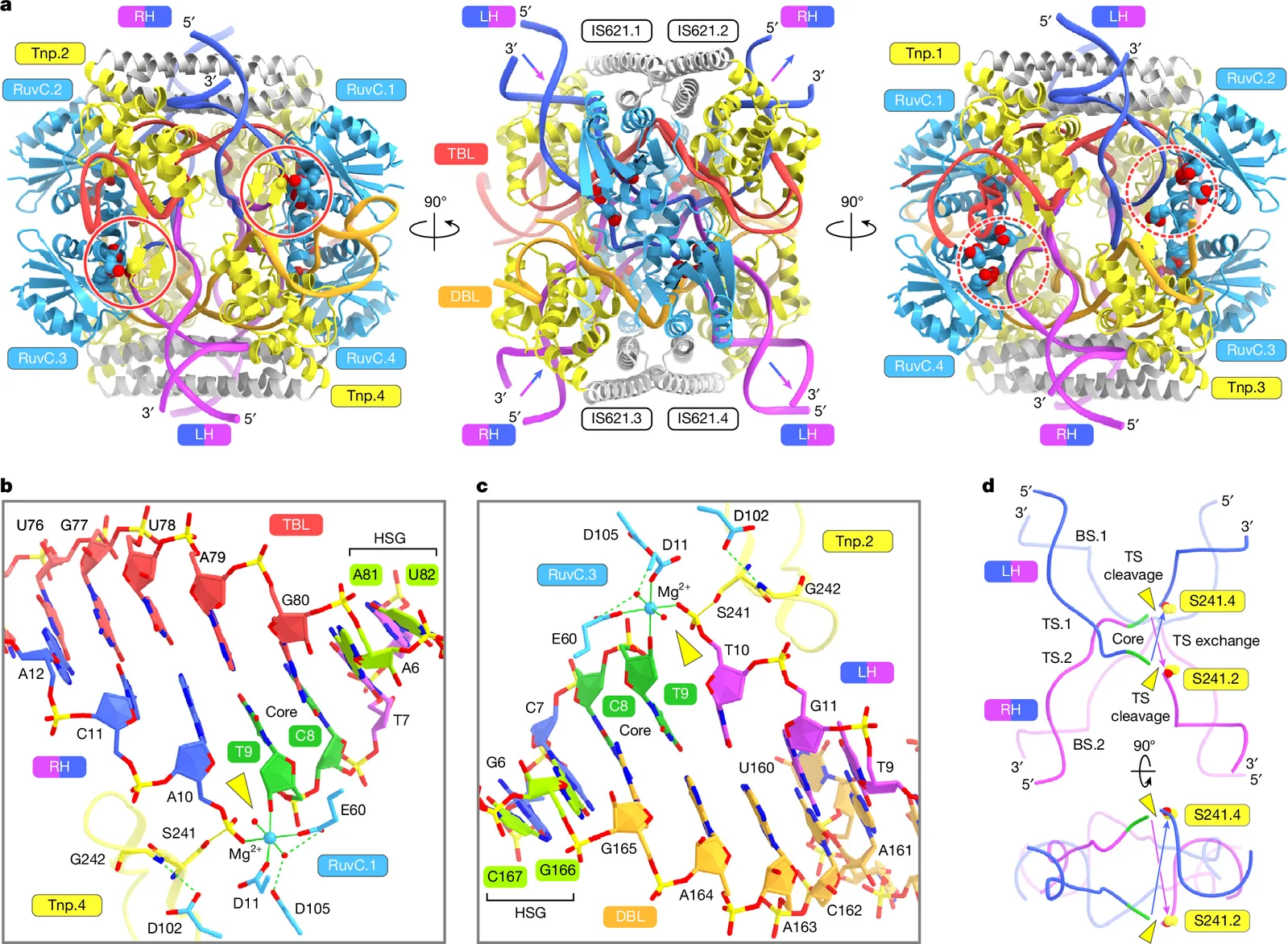
Active site structure. **a**, Locations of the RuvC–Tnp active sites. The RuvC.1–Tnp.4 and RuvC.3–Tnp.2 active sites, containing the ordered S241 residues responsible for the top-strand cleavage, are marked by red circles. The RuvC.4–Tnp.1 and RuvC.2–Tnp.3 active sites, with the disordered S241 residues responsible for the bottom-strand cleavage, are indicated by dashed red circles. **b**,**c**, Close-up views of the RuvC.1–Tnp.4 (**b**) and RuvC.3–Tnp.2 (**c**) active sites. The Mg^2+^ ions and water molecules are shown as cyan and red spheres, respectively. Hydrogen bonds and coordinate bonds are shown as green dashed and solid lines, respectively. **d**, Structures of the DNA substrates after top-strand cleavage. The S241 residues in the Tnp.2 and Tnp.4 domains are shown as space-filling models. Arrows indicate nucleophilic attacks between the 3′-OH groups on the top strands and the 5′-phosphoserine intermediates on the opposite strands. In **b**–**d**, DNA cleavage sites are indicated by yellow triangles.

As in the insertion complex^2^, the top strands of the RH and LH substrates are cleaved adjacent to their CT core motifs (C8–T9) within the composite active sites (RuvC.1–Tnp.4 and RuvC.3–Tnp.2), generating free 3′-OH groups on the T9 nucleotides of both substrates and covalent 5′-phosphoserine intermediates with S241 of Tnp.4 and Tnp.2, respectively (Fig. 3a–c and Supplementary Fig. 2b). Consistently, our in vitro recombination experiments with fluorescently labelled DNA substrates showed the presence of the top-strand-cleaved intermediates during both the excision and insertion reactions (Extended Data Fig. 8b). The top-strand-cleaved intermediates accumulated to similar levels in both reactions, yet the final recombination product was markedly less abundant during excision (Extended Data Fig. 8b). This suggests that strand exchange, rather than initial cleavage, is the rate-limiting step for excision and accounts for much of the efficiency difference between the two reactions. Together, these data indicate that the top strands of the RH and LH substrates are cleaved adjacent to the CT core, forming 5′-phosphoserine intermediates and 3′-OH groups, which enable subsequent strand exchange (Fig. 3d).

## Post-TSE excision complex structure

**Fig. 4:**
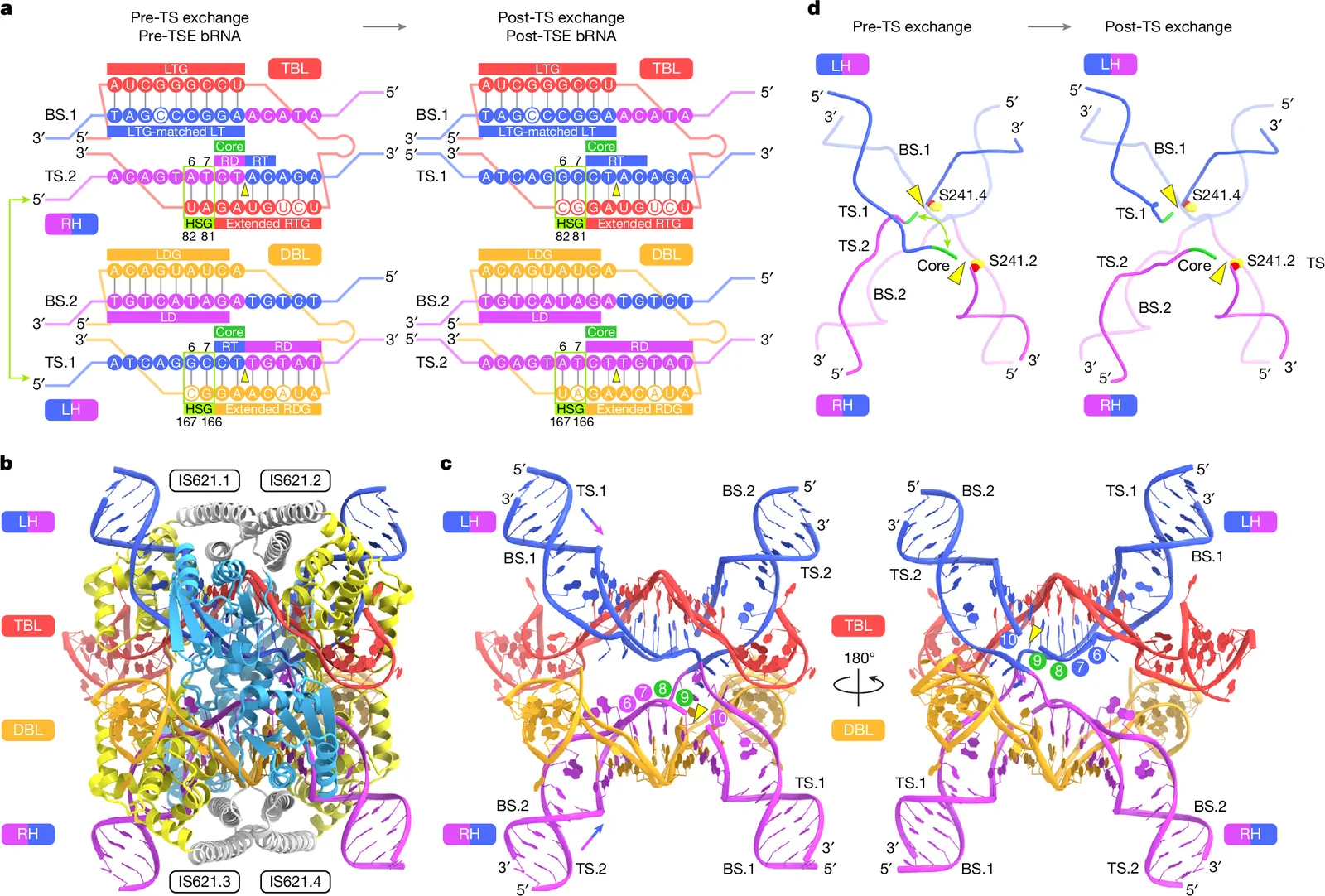
Cryo-EM structure of the IS621 excision complex in the post-strand exchange state. **a**, Schematic of base pairing between the engineered bRNA and the excision substrates before and after top-strand exchange. Replaced nucleotides compared with the wild-type bRNA and DNA are indicated with white backgrounds. HSG nucleotides are highlighted within yellow-green boxes. **b**,**c**, Structures of the IS621–bRNA–LH–RH complex (**b**) and the bRNA–LH–RH complex (**c**) in the post-strand exchange state. Nucleotides surrounding the CT core (positions 6–10) are numbered, with the CT core (positions 8–9) highlighted in green. The directionalities of the LH and RH are indicated by arrows. **d**, Structural comparison of the DNA substrates in the pre-TSE and post-TSE states during excision. The S241 residues in the Tnp.2 and Tnp.4 domains are shown as space-filling models. The top-strand exchange is indicated by a yellow-green arrow. In **a**, **c** and **d**, DNA cleavage sites are indicated by yellow triangles.

To further investigate the excision mechanism, we determined the 2.6-Å resolution cryo-EM structure of the excision complex in the post-strand exchange state, using the engineered bRNA with the post-TSE HSGs (A81G/U82C/G166A/U167), which facilitate HSG–DNA base pairing after top-strand exchange (Fig. 4a–c, Supplementary Fig. 4a–e and Supplementary Video 2). As expected, the top strands of the LH and RH are cleaved adjacent to the CT core and exchanged, forming new TBL–LH and DBL–RH base pairs (Fig. 4a–d). After exchange, the RDG retains its base pairing with positions 10–14 of the LH top strand, while forming new base pairs with the incoming core (C8–T9) of the RH top strand (Fig. 4a,c). Immediately adjacent to this core interaction, the HSG region (A166–U167) of the DBL base pairs with T7–A6 of the incoming RH strand, stabilizing the post-strand exchange state. Similarly, the RTG remains bound to positions 10–14 of the RH top strand, while forming new base pairs with the incoming core (C8–T9) of the LH top strand, with the HSG region of the TBL (G81–C82) base pairing with C7–G6 of the LH (Fig. 4a,c). These structural observations indicate that, similar to the insertion reaction^2^, the top strands of the DNA substrates are exchanged after strand cleavage during the excision reaction.

## Discussion

**Fig. 5:**
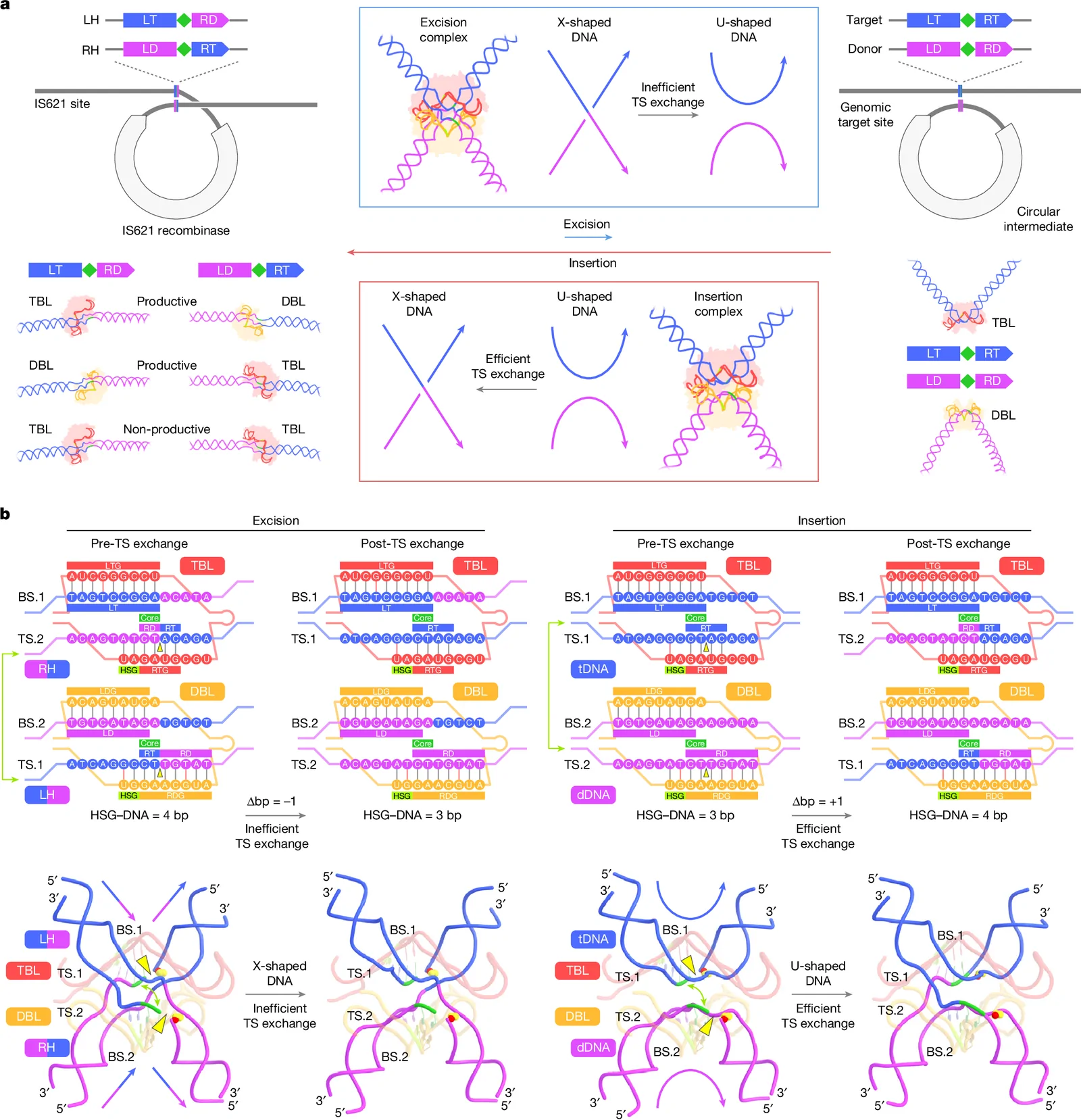
Mechanisms of IS621 element excision and insertion. **a**, Schematic of IS621 element excision and insertion. Different efficiencies of excision and insertion (measured by in vitro assays) are represented by shorter and longer arrows, respectively. **b**, Schematics and structures of the bRNA–DNA complex before and after top-strand exchange in excision and insertion. The directionalities of the DNA substrates are indicated by arrows in the structures. The top-strand exchanges are indicated by yellow-green arrows. DNA cleavage sites are marked with yellow triangles.

In this study, we showed that bRNA is expressed at low levels from linear IS621 elements in *E. coli* cells and that the IS621 recombinase–bRNA complex mediates the recombination between the two junction sequences flanking the element, the LH (LT–RD) and RH (LD–RT), enabling the excision of the IS621 element from the genome (Fig. 5a). Our biochemical data showed that the IS621–bRNA complex catalyses excision with much lower efficiency than insertion. Furthermore, the cryo-EM structures provided insights into how the IS621–bRNA complex recognizes the excision DNA substrates to mediate their recombination. These findings suggest that excision is normally suppressed in *E. coli*, but once a rare excision event occurs, the resulting circular intermediate reconstitutes the RE–LE junction promoter, boosting recombinase and bRNA expression. As the natural system is inherently configured to favour insertion, this would preferentially drive transposition into a new target site.

A structural comparison between the excision complex and the insertion complex^2^ showed mechanistic similarities between the IS621-mediated excision and insertion processes (Fig. 5b, Supplementary Fig. 5 and Supplementary Video 3). Initially, the IS621–bRNA complex recognizes DNA substrates for both excision (LH and RH) and insertion (dDNA and tDNA) primarily through identical RNA–DNA base pairing at four guide regions. In both processes, the top strands of the DNA substrates are cleaved at the CT core within the RuvC–Tnp active sites, forming 5′-phosphoserine intermediates and 3′-OH groups on the top strands and allowing top-strand exchange. It is likely that the bottom strands are subsequently cleaved at the other RuvC–Tnp active sites during excision, as observed during insertion^2^. These structural findings highlight the ability of the IS621 bridge system to mediate programmable recombination by similar but distinct stepwise catalytic mechanisms: excision (that is, recombination within a single double-stranded DNA (dsDNA) molecule with LT–RD and LD–RT) and insertion (that is, recombination between two dsDNA molecules with LT–RT and LD–RD).

Notable mechanistic differences exist between the excision and insertion reactions. First, the DNA substrates adopt different conformations in the two complexes. The insertion complex contains bent, U-shaped tDNA and dDNA substrates, whereas the excision complex binds linear LH and RH substrates, which form an X-shaped structure (Fig. 5a,b and Supplementary Video 3). As the transition from bent to linear conformations in the cleaved DNA top strands is likely energetically favourable, top-strand exchange would occur more efficiently during insertion than during excision, consistent with our biochemical data (Extended Data Fig. 8b). These conformational differences probably contribute to the different efficiencies of the two processes. Moreover, our structural and biochemical data indicate the distinct assembly mechanisms of the IS621–bRNA–DNA tetrameric complex in excision and insertion. During insertion, IS621 recombinase dimers bound to TBL/tDNA and DBL/dDNA assemble into a functional tetrameric complex^2^. By contrast, during excision, tetramers can form through multiple pathways, because TBL- and DBL-bound recombinase dimers can each bind both LH and RH substrates (Extended Data Fig. 10c). Productive excision complexes form when a TBL-bound recombinase dimer engaged with the LH and RH assembles with a DBL-bound recombinase dimer engaged with the RH and LH, respectively (Fig. 5a). However, alternative, non-productive combinations may occur and could reduce the overall excision efficiency. Although two TBL-bound recombinase dimers could simultaneously bind to the LH and RH sequences in the natural IS621 locus, they cannot form a productive complex, preventing recombination and thereby reducing excision in *E. coli* cells (Fig. 5a). Our biochemical data showed that the DBL alone can support excision (Extended Data Fig. 6a), suggesting that recombinase dimers bound to DBL–LH and DBL–RH can form a productive complex, in which the DNA substrates probably adopt U-shaped rather than X-shaped conformations because of a steric clash between the linear LH and RH substrates. Furthermore, in the natural IS621 system, the LH (LT–RD) and RH (LD–RT) sequences are located at both ends of the elements on the same DNA molecule (genomic DNA), whereas the target (LT–RT) and donor (LD–RD) sequences reside on separate DNA molecules (genomic DNA and circular intermediates) (Fig. 5a). These positional differences in the recognition sequences are also likely to affect the efficiencies of the excision and insertion processes.

Typically, RNA-guided enzymes such as CRISPR–Cas9^16^ recognize nucleic-acid targets by base pairing with an RNA guide and cleave them within the guide–target duplex. By contrast, the IS621 bridge recombinase recognizes two DNA substrates by base pairing with the four RNA guide segments in the bRNA, and then a portion of the substrates (positions 6–9 in the top strands) must dissociate from the guides after top-strand cleavage for the subsequent strand exchange (Fig. 5b and Supplementary Fig. 5). As the CT core dinucleotides (positions 8–9 in the top strands) are invariant and always base pair with the guide segments, the extent of the base pairing between the top strand of the DNA substrates at positions 6–7 and the HSG dinucleotides of the bRNA (TBL positions 81–82 and DBL positions 166–167) serves as the primary determinant of the recombination efficiency (Fig. 5b and Supplementary Fig. 5). Our previous study showed that HSG–DNA base pairing before and after strand exchange inhibits and facilitates the insertion reaction, respectively^2^. In this study, we found that HSG–DNA base pairing also affects the efficiency of the excision reaction, highlighting a common mechanistic principle between excision and insertion. The wild-type bRNA contains AU (positions 81–82 in TBL) and GU (positions 166–167 in DBL) as the HSG sequences, whereas the wild-type tDNA and dDNA have GC and AT at positions 6 and 7, respectively (Fig. 5b and Supplementary Fig. 5). The wild-type HSG sequences form only one Watson–Crick base pair with the DNA before strand exchange, but three after exchange. This asymmetry drives the insertion reaction forward while simultaneously disfavouring excision, consistent with our biochemical data. Together, these observations suggest that, apart from the low bRNA expression and the limited excision activity of the IS621 complex, the native HSG–DNA base-pairing pattern substantially contributes to suppressing excision in *E. coli* cells. This base-pairing pattern is highly conserved among other IS110 elements^2^, supporting the role of HSGs as key regulators of the IS110 life cycle.

This study clarifies the programmable recombination mechanism of IS110 bridge systems and shows that the optimal bRNA designs for insertion are inherently unfavourable for excision—a principle essential for maximizing the persistence of genome modifications. The engineering principles presented here offer a practical guide for optimizing bRNA scaffolds to favour either insertion or excision. Several modifications, including the split bRNA system and RTG/RDG extensions, have already improved recombination efficiency in human cells^12,13^, underscoring the translational relevance of our findings.

## Methods

### RNA sequencing and analysis

*E. coli* BL21(DE3) cells (NEB: C2527H), which lack endogenous copies of IS621 elements, were transformed with plasmids encoding either the RE–LE junction or the LE–recombinase–RE region of the IS621 element. The cells were plated on LB (lysogeny broth) agar with kanamycin and grown overnight at 37 °C. Colonies were scraped from the plates, and RNA was extracted using a Direct-zol RNA miniprep kit (Zymo Research). To assess the expression of the IS621 elements encoded in the *E. coli* genome, RNA was extracted using a Direct-zol RNA miniprep kit from One Shot *E. coli* Mach1 T1^R^ cells (F– φ80*lac*ZΔM15 Δ*lac*X74 *hsd*R(*r*_*K–*_, *m*_*K+*_) Δ*rec*A1398 *end*A1 *ton*A) (Thermo Fisher Scientific, C862003) harvested from bacterial plates 24 h after plating at 37 °C.

RNA-seq was carried out on a NovaSeq X Plus sequencer in collaboration with Novogene Research Services (Novogene). Paired-end RNA-seq reads from each sample were aligned to the respective plasmid and genomic reference sequences using Burrows–Wheeler Aligner-MEM^17^. Aligned reads were filtered using SAMTools^18^ for reads that originated from the strand that encodes the bRNAs. For each locus of interest on the reference sequences, we used the Pysam Python module to count the coverage depth at the single-base level from these filtered reads, with a quality threshold of 0. As the *E. coli* Mach1 genome has three separate IS621 loci that are highly homologous with one another, many of the RNA-seq reads that mapped to these regions were expected to lack unique mapping to one of the three loci. Thus, among the reads that were mapped to any of the IS621 loci, pairs of reads in which either pair had a mapping quality of zero (meaning the read can map equally well to other parts of the reference sequence) were classified as ‘nonspecific’ reads, and the coverage from these reads was counted separately from the reads that mapped specifically to one of the three IS621 loci.

### Detection of plasmid-based IS621 excision by PCR

Plasmids containing elements with the wild-type bRNA (pre-TSE for excision) or the engineered bRNA (post-TSE for excision) were transformed into *E. coli* BL21(DE3), which lacks endogenous copies of IS621 elements, ensuring that all detected excision events originate from the plasmid-borne construct. For constructs lacking a T7 promoter upstream of the bRNA, bacteria were plated on LB agar with kanamycin and grown overnight at 37 °C. For constructs with a T7 promoter, plates were supplemented with 0.07 mM IPTG. Colonies were scraped from plates, and plasmid DNA was extracted using a QIAprep Spin Miniprep Plus Kit (Qiagen). Primers were designed to span the post-excision (LT–RT) junction site left after element excision from the plasmid and yield a 110-bp product. The same primers used to detect circularized IS621 intermediates from the Mach1 genome were also used to confirm the production of circular intermediates from plasmid-borne constructs, yielding a 725-bp product. All PCRs were performed with the same protocol described below for the detection of native IS621 excision and were fractionated on 2% agarose gels and visualized with SYBR Gold (Thermo Fisher Scientific). All PCR products were sequence-verified by Sanger sequencing.

### Detection of circular intermediates and post-excision sites

Genomic DNA was isolated from freshly purchased aliquots of *E. coli* strains in liquid culture using Zymo Quick DNA Miniprep Plus Kits (Zymo), according to the instructions of the manufacturer. Whole-genome sequencing was performed at 100× coverage using the Plasmidsaurus whole-genome nanopore sequencing service, which confirmed that the One Shot Mach1 *E. coli* T1^R^ strain harbours three copies of IS621, whereas the *E. coli* BL21(DE3) strain contains zero copies. Bacteria were plated on LB agar and grown overnight at 37 °C. Colonies were then scraped from the plates, and total DNA was extracted using a Zymo Quick DNA Miniprep Plus Kit. Primers were designed to match all three IS621 recombinase-coding sequences in the Mach1 genome, such that PCR amplification would occur only on excised and circularized IS621 sequences from any locus. Moreover, primers were designed to base pair with the junction remaining post-excision at all three loci encoding IS621, such that amplification with a downstream primer would yield a specific product. All PCRs were performed using Platinum SuperFi 2× MasterMix (Thermo Fisher Scientific) with the following protocol: 98 °C for 120 s; 25 cycles of 98 °C for 10 s, 65 °C for 15 s and 72 °C for 15 s; and 72 °C for 2 min. PCR products were subsequently fractionated on 2% agarose gels and visualized with SYBR Gold.

### In vitro recombination measurement by qPCR

DNA substrates (51-bp LH, 121-bp RH, 86-bp tDNA and 86-bp dDNA) were purchased from IDT as ssDNA and annealed by heating to 95 °C followed by slow cooling to 4 °C over 1 h in a thermocycler. DNA substrates (0.25–0.5 µM) were mixed with the IS621–bRNA complex (10 µM) in 20 µl buffer containing 20 mM Tris-HCl, pH 7.5, 300 mM NaCl, 5 mM MgCl_2_ and 1 mM dithiothreitol (DTT), and then the reactions were incubated at 37 °C for 2 h. The reactions were quenched by the addition of 50 mM EDTA, treated with 8 µg RNase A (NEB) at 50 °C for 1 h, and then treated with 3 units of Proteinase K at 37 °C for 1 h. After RNA and protein digestion, the DNA was purified using DNA Clean & Concentrator-5 (Zymo Research) and eluted with 75 °C nuclease-free water. For qPCR analysis, reaction dilutions were prepared, and qPCR was performed on a LightCycler 480 II instrument (Roche) using primers and PrimeTime qPCR probes (final concentration 0.5 µM) purchased from IDT, along with TaqMan Fast Advanced qPCR mix. In vitro recombination products are expected to have an unligated bottom strand, with the two nucleotides adjacent (3′) to the core mismatched with the top strand, after the bottom strand exchange step of the reaction mechanism. Therefore, to accurately quantify the reaction efficiency, considering the polymerase processivity through lesions and the primer binding near nicks, qPCR signals for different reactions were compared with standard curves generated using known quantities of 121-bp RH (LD–RT, insertion product) DNA and 86-bp target (LT–RT, excision product) DNA containing an unligated bottom strand with the expected mismatches adjacent to the core. These standards were prepared by annealing the top strand of RH/target (121/86 nt) with two oligos (56- and 65-nt RH and 56- and 30-nt target) constituting the bottom strand.

### In vivo excision efficiency measurement by qPCR

To determine the excision efficiency in *E. coli* using the wild-type (pre-TSE for excision) and the engineered bRNA (post-TSE for excision) encoded within the LE of the IS621 element, 100 ng of sequence-verified plasmid, confirmed by nanopore sequencing to lack pre-excised molecules in the input population, was transformed into chemically competent BL21(DE3) cells, which were recovered in SOC (super optimal broth) medium for 1 h at 37 °C, plated onto kanamycin-selective agar and grown overnight at 37 °C. All colonies from a single transformation plate were bulk-harvested by scraping into liquid medium, providing a population-level average of excision efficiency across thousands of independent clonal lineages and minimizing bias from stochastic colony-to-colony variation. Total plasmid DNA was extracted from each pooled sample using a QIAprep Spin Miniprep Plus Kit (Qiagen), and qPCR was carried out on 400 ng of the extracted plasmid. Primers and probes were designed to amplify the KanR resistance marker (HEX) as a measure of total plasmid, as well as the post-excision junction (LT–RT, FAM) on plasmids that had undergone excision of the IS621 element. Multiplexed TaqMan qPCR was performed on a control plasmid containing both the post-excision junction and the KanR marker to determine the relative amplification efficiencies of the two probes. This inter-channel correction factor was applied when calculating the excision efficiency from experimental samples. At least three independent transformations were performed to confirm the reproducibility of the population-level measurement.

### Protein and RNA preparation

The IS621 recombinase protein was prepared as described previously^2^. Briefly, the IS621 gene was cloned into a modified pFastBac1 vector encoding an N-terminal His_6_–Twin-Strep tag and an HRV3C cleavage site. Sf9 cells were infected with the baculovirus and cultured at 27 °C for 48 h. The cells were harvested and lysed, and the soluble fraction was purified by Strep-Tactin affinity chromatography followed by size-exclusion chromatography. The purified protein was concentrated and stored at −80 °C, in buffer containing 20 mM HEPES-NaOH, pH 7.5, 500 mM NaCl, 2 mM MgCl_2_, 1 mM DTT and 10% glycerol, until use. The bRNAs were transcribed in vitro with T7 RNA polymerase and purified by 10% denaturing (7 M urea) polyacrylamide gel electrophoresis (Supplementary Table 1).

### In vitro recombination assays with fluorescently labelled DNA substrates

DNA substrates (38-bp LH, 44-bp RH, 38-bp tDNA and 44-bp dDNA) were purchased from Eurofins Genomics. The LH and tDNA were labelled with Cy5 at the 5′ ends of the top strand (Supplementary Table 1). The DNA substrates (0.4 µM; LH and RH for excision or tDNA and dDNA for insertion) were mixed with the pre-incubated IS621–bRNA complex (2 μM) in 10 µl buffer containing 20 mM Tris-HCl, pH 7.5, 300 mM NaCl, 5 mM MgCl_2_ and 1 mM DTT, and then the reactions were incubated at 37 °C for 1 h. The reaction mixture was mixed with Proteinase K (Nacalai Tesque) and incubated at 95 °C for 2 min in denaturing buffer (7 M urea). The samples were analysed on an 18% TBE–urea denaturing gel, and fluorescent signals were imaged using FUSION Solo S (Vilber Bio Imaging).

### Excision complex preparation

The excision complex was reconstituted by mixing the purified IS621 recombinase, a 177-nt bRNA (177 nucleotides plus a 5′ GGG leader for in vitro transcription) and DNA substrates (LH and RH) containing 4-nt mismatches on the top strands. The bRNAs with pre-TSE and post-TSE were used for reconstitution of the excision complex in the pre- and post-strand exchange states, respectively. The reconstituted complex was purified by size-exclusion chromatography on a Superose 6 Increase 10/300 column (Cytiva), equilibrated with buffer (20 mM Tris-HCl, pH 7.5, 300 mM NaCl, 5 mM MgCl_2_ and 1 mM DTT). The purified excision complex was concentrated to 0.5–1 mg ml^−1^ using an Amicon Ultra-4 centrifugal filter unit (MWCO 50 kDa; Millipore). Protein concentrations were determined using the Pierce 660 nm Protein Assay Reagent.

### Cryo-EM analysis

For cryo-EM data collection, Quantifoil Holey Carbon Grids (R1.2/1.3, Au, 300 mesh) (SPT Labtech) were glow-discharged in low-pressure air at a 10 mA current in a PIB-10 (Vacuum Device). The excision complex solution was applied to freshly glow-discharged grids using a Vitrobot Mark IV system (Thermo Fisher Scientific) at 4 °C, with a waiting time of 10 s and a blotting time of 6 s under 100% humidity conditions. The grids were plunge-frozen in liquid ethane cooled by liquid nitrogen.

The grids were transferred to a Titan Krios G3i TEM (Thermo Fisher Scientific), running at 300 kV and equipped with a Gatan Quantum-LS Energy Filter (GIF) and a Gatan K3 Summit direct electron detector. Imaging was performed at a nominal magnification of 105,000×, corresponding to a calibrated pixel size of 0.83 Å per pixel (px). Each movie was dose-fractionated to 50 frames at a dose rate of 7.8 e^−^ px^−1^ s^−1^ at the detector in the correlated double sampling mode, resulting in a total accumulated exposure of 50 e^−^ Å^−2^ of the specimen. The data were automatically acquired using the image-shift method in the EPU software (Thermo Fisher Scientific), with a defocus range of −0.8 μm to −2.0 μm.

The data were processed using the cryoSPARC v.4.4.0 software package^19^. The dose-fractionated movies were aligned using Patch Motion Correction, and the contrast transfer function (CTF) parameters were estimated using patch-based CTF estimation. For the excision complex with the pre-TSE bRNA, particles were automatically picked using Blob Picker and Template Picker, followed by reference-free two-dimensional classification to curate particle sets. The particles were further curated by several cycles of Heterogeneous Refinement. The best class particle set was refined using Homogeneous Refinement, yielding a map at 2.71-Å resolution. Reference-Based Motion Correction followed by Non-Uniform Refinement with optimization of the CTF value yielded a map at 2.44 Å resolution (according to the Fourier shell correlation (FSC) = 0.143 criterion)^20^ using a tight mask; however, the final reported resolution was determined to be 2.53 Å based on the FSC calculated using the softer refinement mask. For the excision complex with the post-TSE bRNA, particles were automatically picked using Template Picker followed by reference-free two-dimensional classification. The particles were further curated by several cycles of Heterogeneous Refinement. The best class particle set was refined using Non-Uniform Refinement, yielding a map at 2.62 Å resolution. Reference-Based Motion Correction followed by Non-Uniform Refinement with optimization of the CTF value yielded a map at 2.41 Å resolution (according to the FSC = 0.143 criterion) using a tight mask; however, the final reported resolution was determined to be 2.55 Å based on the FSC calculated using the softer refinement mask. The local resolution was estimated by BlocRes in cryoSPARC.

### Model building and validation

The models of the excision complexes were manually built with COOT^21^, using the IS621 insertion complex model (PDB ID: 8WT6) as the initial model, and then refined using Servalcat^22^ against unsharpened half-maps. The models were validated using MolProbity^23^. The statistics of the three-dimensional reconstruction and model refinement are summarized in Extended Data Table 1. The cryo-EM density maps were calculated with UCSF ChimeraX^24^, and molecular graphics figures were prepared with CueMol (http://www.cuemol.org).

### Microscale thermophoresis

Microscale thermophoresis (MST) was performed using a Monolith NT.115 Pico Series instrument (NanoTemper Technologies) with premium capillaries. The IS621 recombinase was labelled using a RED-MALEIMIDE 2nd Generation cysteine-reactive kit (NanoTemper Technologies), according to the instructions of the manufacturer. The labelled protein was diluted in buffer containing 20 mM Tris-HCl, pH 7.5, 500 mM NaCl, 5 mM MgCl_2_, 1 mM DTT and 0.01% Tween 20, and ligands were prepared by dilution in the same buffer. For ligand preparation, DNA was purchased from IDT and annealed in buffer containing 10 mM Tris, pH 8.0, 5 mM MgCl_2_ and 5 mM KCl. To determine the affinities of the IS621–RNA complex for the DNA substrates (tDNA, dDNA, LH and RH), the IS621–RNA complex (20 nM) was incubated with serial dilutions (0.3 nM to 10 µM) of each relevant DNA ligand. MST measurements were performed at 37 °C with 8% LED excitation and medium MST power in the Pico-RED excitation mode. Data were analysed using the NanoTemper MO.affinity analysis software package, and raw data were plotted on Prism for visualization.

### Reporting summary

Further information on research design is available in the Nature Portfolio Reporting Summary linked to this article.

## Online content

Any methods, additional references, Nature Portfolio reporting summaries, source data, extended data, supplementary information, acknowledgements, peer review information; details of author contributions and competing interests; and statements of data and code availability are available at https://doi.org/10.1038/s41586-026-10903-y.

## Supplementary information

Supplementary Information This file contains Supplementary Figs. 1–6.

Reporting Summary

Supplementary Table 1 Nucleic acids used in this study.

Peer Review File

Supplementary Video 1 Structure of the IS621 excision complex in the pre-top strand exchange state. The active-site residues are shown as stick models, with the core and HSG nucleotides coloured green and yellow-green, respectively.

Supplementary Video 2 Structure of the IS621 excision complex in the post-top strand exchange state. The active-site residues are shown as stick models, with the core and HSG nucleotides coloured green and yellow-green, respectively.

Supplementary Video 3 Structural comparison between the IS621 excision and insertion complexes in the pre- and post-top strand exchange states. Nucleobases involved in RNA–DNA base pairing are shown as stick models, with core nucleotides coloured green and HSG nucleotides coloured yellow-green. The recombinase molecules are omitted for clarity, whereas the S241 residues in the Tnp.2 and Tnp.4 domains are shown as space-filling models.

## Figures and Tables

**Extended Data Table 1:**
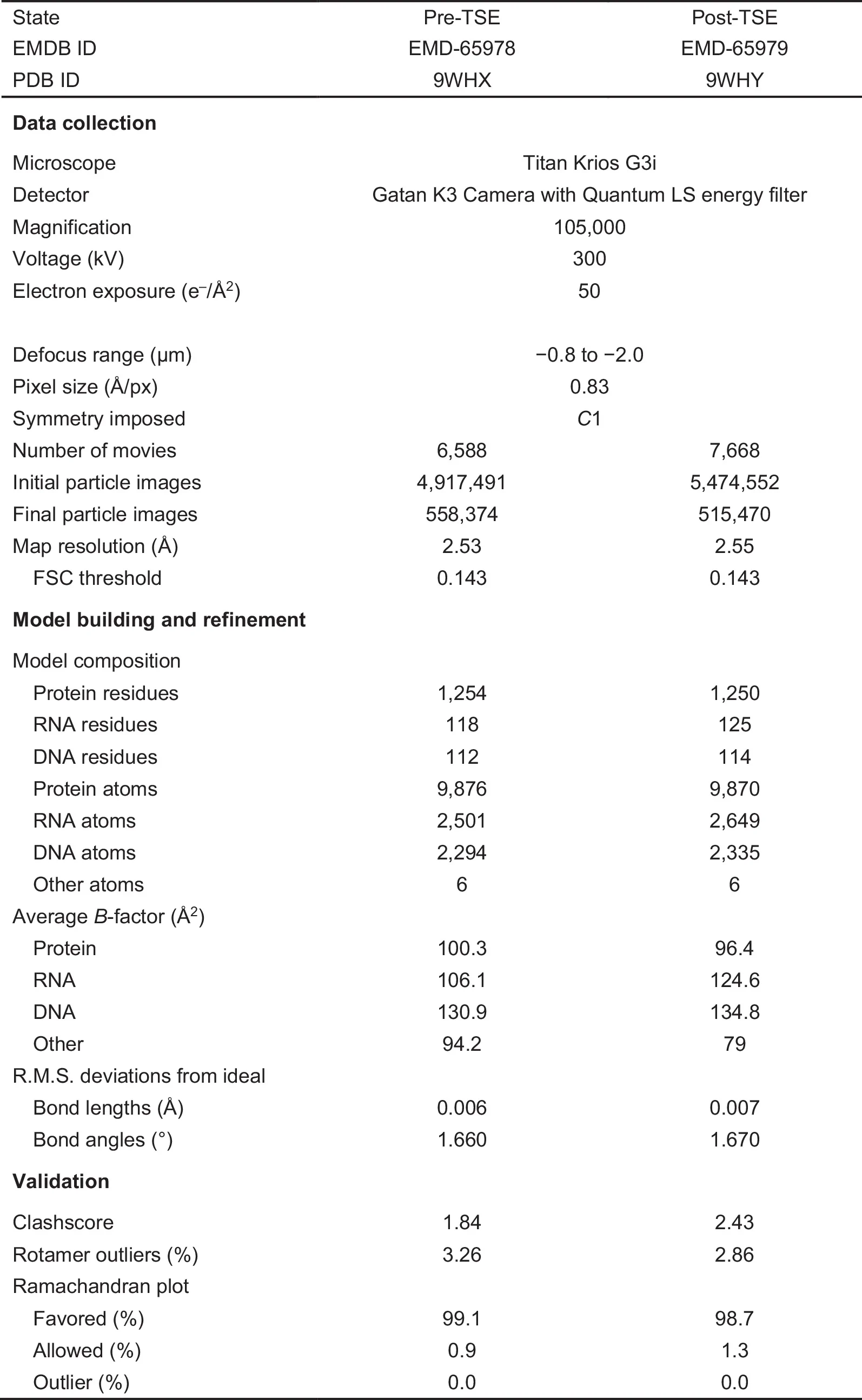
Cryo-EM data collection, refinement, and validation statistics

## Data Availability

Cryo-EM density maps have been deposited in the Electron Microscopy Data Bank under the accession codes EMD-65978 (pre-strand exchange state) and EMD-65979 (post-strand exchange state). Atomic coordinates have been deposited in the Protein Data Bank under IDs 9WHX (pre-strand exchange state) and 9WHY (post-strand exchange state). The raw images have been deposited in the Electron Microscopy Public Image Archive, under the accession code EMPIAR-13446. The RNA-seq data and the *E. coli* Mach1-T1R chromosome assembly used as the reference for RNA-seq alignment in this study have been deposited under NCBI BioProject accession PRJNA1455502. The Mach1-T1R chromosome assembly can also be accessed using GenBank accession number JCAHTY000000000, and the RNA-seq data using the Gene Expression Omnibus (GEO) database under accession number GSE328474. The Mach1-T1R strain was obtained from Thermo Fisher Scientific (cat. no. C862003).

## References

[1] Durrant, M. G. et al. Bridge RNAs direct programmable recombination of target and donor DNA. *Nature* **630**, 984–993 (2024). 10.1038/s41586-024-07552-4 PMC11208160 38926615

[2] Hiraizumi, M. et al. Structural mechanism of bridge RNA-guided recombination. *Nature* **630**, 994–1002 (2024). 10.1038/s41586-024-07570-2 PMC11208158 38926616

[3] Siguier, P., Gourbeyre, E., Varani, A., Ton-Hoang, B. & Chandler, M. Everyman’s guide to bacterial insertion sequences. *Microbiol. Spectr*. 10.1128/microbiolspec.mdna3-0030-2014 (2015). 26104715

[4] Perkins-Balding, D., Duval-Valentin, G. & Glasgow, A. C. Excision of IS*492* requires flanking target sequences and results in circle formation in *Pseudoalteromonas atlantica*. *J. Bacteriol.* **181**, 4937–4948 (1999). 10.1128/jb.181.16.4937-4948.1999 PMC93982 10438765

[5] Müller, C., Lauf, U. & Hermann, H. The inverted repeats of IS*1384*, a newly described insertion sequence from *Pseudomonas putida* strain H, represent the specific target for integration of IS*1383*. *Mol. Genet. Genomics* **265**, 1004–1010 (2001). 10.1007/s004380100495 11523772

[6] Choi, S., Ohta, S. & Ohtsubo, E. A novel IS element, IS*621*, of the IS*110*/IS*492* family transposes to a specific site in repetitive extragenic palindromic sequences in *Escherichia coli*. *J. Bacteriol.* **185**, 4891–4900 (2003). 10.1128/JB.185.16.4891-4900.2003 PMC166490 12897009

[7] Partridge, S. R. & Hall, R. M. The IS*1111* family members IS*4321* and IS*5075* have subterminal inverted repeats and target the terminal inverted repeats of Tn*21* family transposons. *J. Bacteriol.* **185**, 6371–6384 (2003). 10.1128/JB.185.21.6371-6384.2003 PMC219399 14563872

[8] Prosseda, G. et al. Plasticity of the P_junc_ promoter of ISEc*11*, a new insertion sequence of the IS*1111* family. *J. Bacteriol.* **188**, 4681–4689 (2006). 10.1128/JB.00332-06 PMC1483014 16788177

[9] Higgins, B. P., Carpenter, C. D. & Karls, A. C. Chromosomal context directs high-frequency precise excision of IS*492* in *Pseudoalteromonas atlantica*. *Proc. Natl Acad. Sci. USA* **104**, 1901–1906 (2007). 10.1073/pnas.0608633104 PMC1794265 17264213

[10] Higgins, B. P., Popkowski, A. C., Caruana, P. R. & Karls, A. C. Site-specific insertion of IS*492* in *Pseudoalteromonas atlantica*. *J. Bacteriol.* **191**, 6408–6414 (2009). 10.1128/JB.00771-09 PMC2753022 19684137

[11] Curcio, M. J. & Derbyshire, K. M. The outs and ins of transposition: from Mu to Kangaroo. *Nat. Rev. Mol. Cell Biol.* **4**, 865–877 (2003). 10.1038/nrm1241 14682279

[12] Perry, N. T. et al. Megabase-scale human genome rearrangement with programmable bridge recombinases. *Science* **391**, eadz0276 (2026). 10.1126/science.adz0276 40997214

[13] Pelea, O. et al. Programmable genome editing in human cells using RNA-guided bridge recombinases. *Science* **391**, eadz1884 (2026). 10.1126/science.adz1884 41642947

[14] Siddiquee, R., Pong, C. H., Hall, R. M. & Ataide, S. F. A programmable seekRNA guides target selection by IS*1111* and IS*110* type insertion sequences. *Nat. Commun.* **15**, 5235 (2024). 10.1038/s41467-024-49474-9 PMC11187229 38898016

[15] Parés-Guillén, E., Yuste, L., Rojo, F. & Moreno, R. The ISPpu9 insertion sequence of *Pseudomonas putida* KT2440 generates various circular intermediates enabling modular transposition. *Nucleic Acids Res.* **53**, gkaf668 (2025). 10.1093/nar/gkaf668 PMC12288879 40705920

[16] Jinek, M. et al. A programmable dual-RNA-guided DNA endonuclease in adaptive bacterial immunity. *Science* **337**, 816–821 (2012). 10.1126/science.1225829 PMC6286148 22745249

[17] Li, H. et al. The sequence alignment/map format and SAMtools. *Bioinformatics* **25**, 2078–2079 (2009). 10.1093/bioinformatics/btp352 PMC2723002 19505943

[18] Danecek, P. et al. Twelve years of SAMtools and BCFtools. *GigaScience* **10**, giab008 (2021). 10.1093/gigascience/giab008 PMC7931819 33590861

[19] Punjani, A., Rubinstein, J. L., Fleet, D. J. & Brubaker, M. A. cryoSPARC: algorithms for rapid unsupervised cryo-EM structure determination. *Nat. Methods* **14**, 290–296 (2017). 10.1038/nmeth.4169 28165473

[20] Rosenthal, P. B. & Henderson, R. Optimal determination of particle orientation, absolute hand, and contrast loss in single-particle electron cryomicroscopy. *J. Mol. Biol.* **333**, 721–745 (2003). 10.1016/j.jmb.2003.07.013 14568533

[21] Emsley, P., Lohkamp, B., Scott, W. G. & Cowtan, K. Features and development of Coot. *Acta Crystallogr. D Biol. Crystallogr.* **66**, 486–501 (2010). 10.1107/S0907444910007493 PMC2852313 20383002

[22] Yamashita, K., Palmer, C. M., Burnley, T. & Murshudov, G. N. Cryo-EM single-particle structure refinement and map calculation using *Servalcat*. *Acta Crystallogr. D Struct. Biol.* **77**, 1282–1291 (2021). 10.1107/S2059798321009475 PMC8489229 34605431

[23] Williams, C. J. et al. MolProbity: more and better reference data for improved all-atom structure validation. *Protein Sci.* **27**, 293–315 (2018). 10.1002/pro.3330 PMC5734394 29067766

[24] Pettersen, E. F. et al. UCSF ChimeraX: structure visualization for researchers, educators, and developers. *Protein Sci.* **30**, 70–82 (2021). 10.1002/pro.3943 PMC7737788 32881101

[25] Abramson, J. et al. Accurate structure prediction of biomolecular interactions with AlphaFold 3. *Nature* **630**, 493–500 (2024). 10.1038/s41586-024-07487-w PMC11168924 38718835

