# Supplementary material for: Structural mechanism governing the directionality of bridge recombination
Source: Nature. 2026 Aug 12;658(8134):269–78. doi: 10.1038/s41586-026-10903-y (PMC13626964; doi:10.1038/s41586-026-10903-y)
Supplement: Supplementary file 1 [file 41586_2026_10903_MOESM1_ESM.pdf]

nature portfolio

https://doi.org/10.1038/s41586-026-10903-y

Supplementary information

Structural mechanism governing the directionality of bridge recombination

In the format provided by the authors and unedited

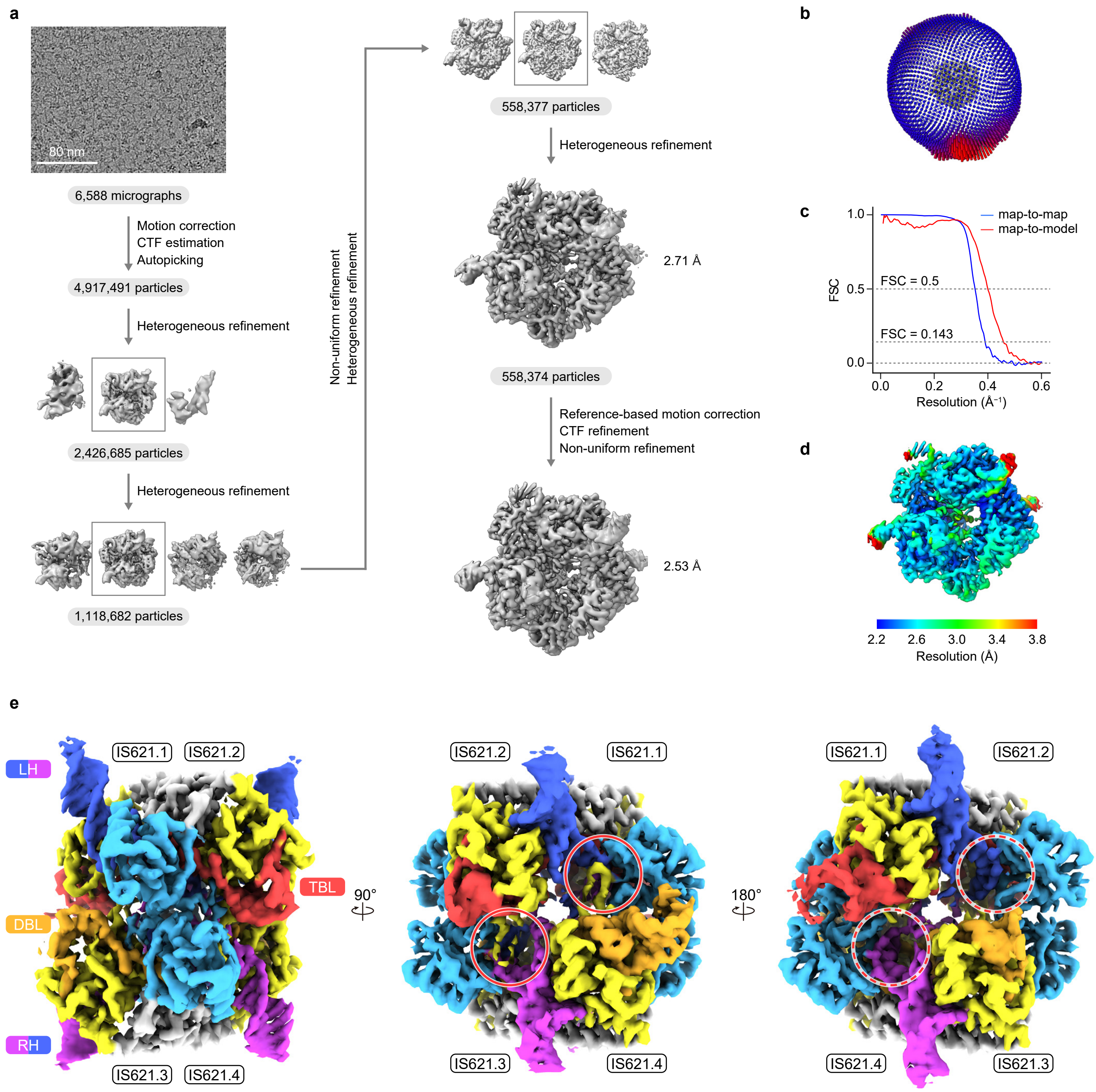

Supplementary Fig. 1 | Cryo-EM analysis of the IS621 excision complex in the pre-strand exchange state.

a, Single-particle cryo-EM image processing workflow.

b, Angular distribution of particles in the final reconstruction.

c, Fourier shell correlation (FSC) curves. The map-to-map FSC curve was calculated between the two independently refined half-maps after masking (blue line), and the overall resolution was determined by the gold standard FSC = 0.143 criterion. The map-to-model FSC curve was calculated between the refined atomic models and maps (red line).

d,**e**, Cryo-EM density maps, colored according to the local resolution (**d**) and the protein domains (**e**).

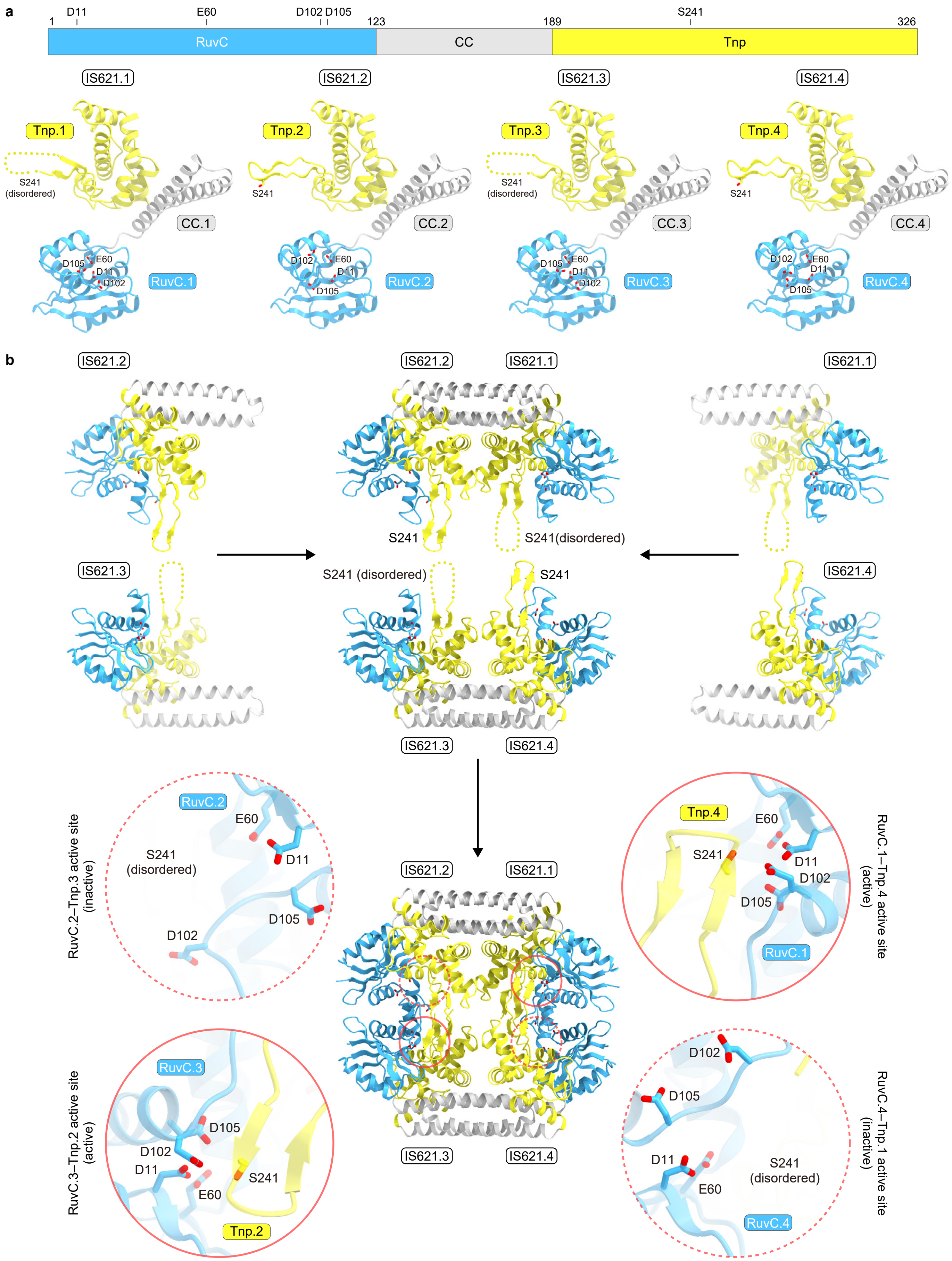

Supplementary Fig. 2 | IS621 recombinase structure.

a, Structures of the four IS621 protomers. The disordered S241 loops in IS621.1 and IS621.3 are indicated by dotted lines. The active-site residues (D11, E60, D102, D105, and S241) are depicted as stick models.

b, Dimeric and tetrameric assemblies of the IS621 recombinase. The RuvC.1–Tnp.4 and RuvC.3–Tnp.2 active sites, containing the ordered S241 residues responsible for the top-strand cleavage, are marked by red circles. The RuvC.4–Tnp.1 and RuvC.2–Tnp.3 active sites, with the disordered Ser241 residues responsible for the bottom-strand cleavage, are indicated by dashed red circles.

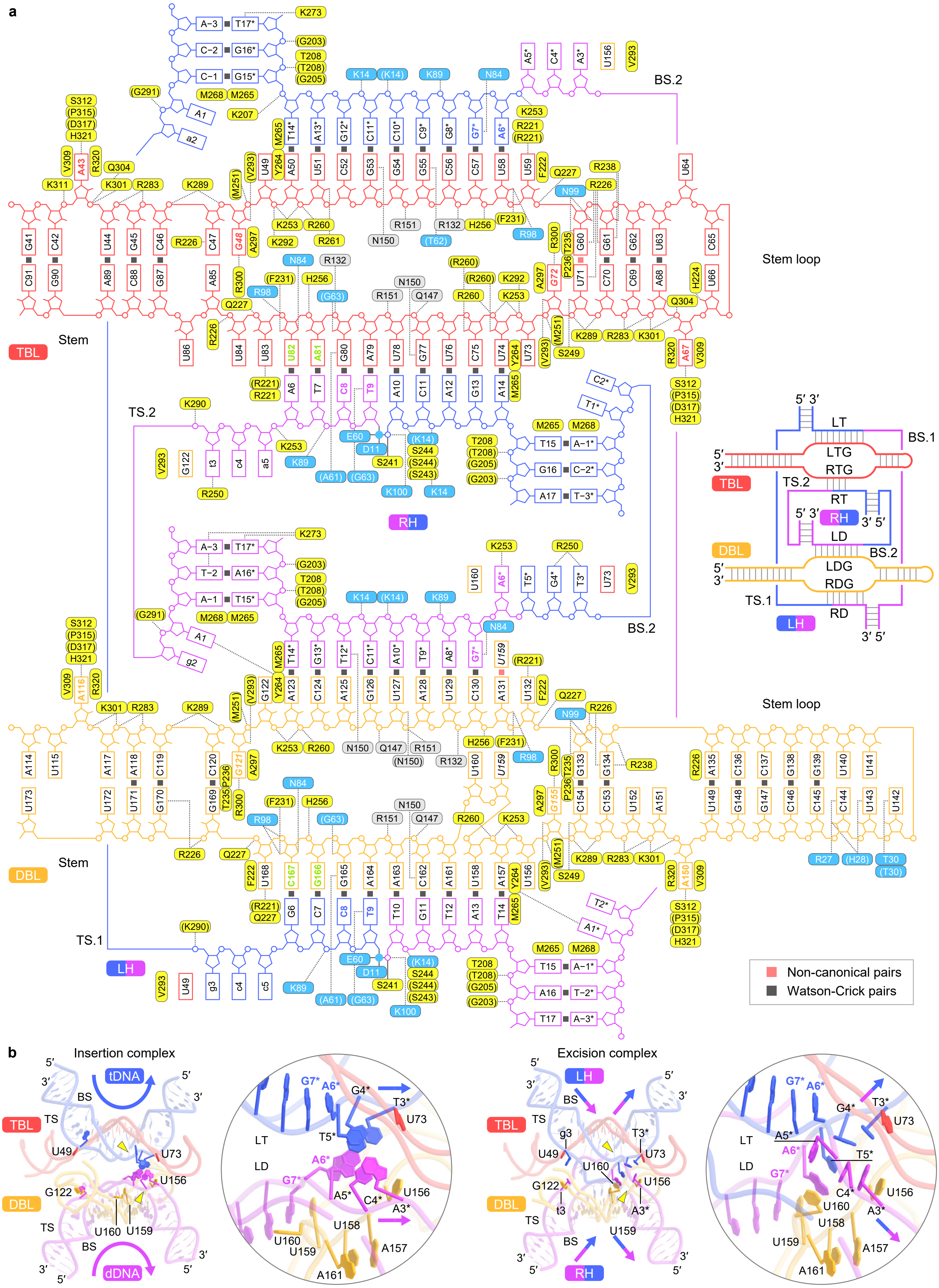

Supplementary Fig. 3 | Recognition of the bRNA and DNA substrates.

a, Schematic showing interactions between the IS621 recombinase and nucleic acids. Conserved nucleotides in the TBL and DBL (A43/A67/A116/A150 and G48/G72/G121/G155) and the core nucleotides (C8, T9, A6*, and G7*) are highlighted in bold. G48/G72/G121/G155 and U159, which adopt the *syn* conformation, are shown in italics. The Mg^{2+} ions are depicted as cyan circles. Non-canonical base pairs are indicated by red squares. The IS621 residues that interact with the nucleic acids through their main chains are shown in parentheses.

b, Structural comparison of the bRNA–DNA complexes in the excision and insertion (PDB: 8WT7) complexes in the pre-strand exchange states. The directionalities of the DNA substrates are indicated by arrows. DNA cleavage sites are marked with yellow triangles.

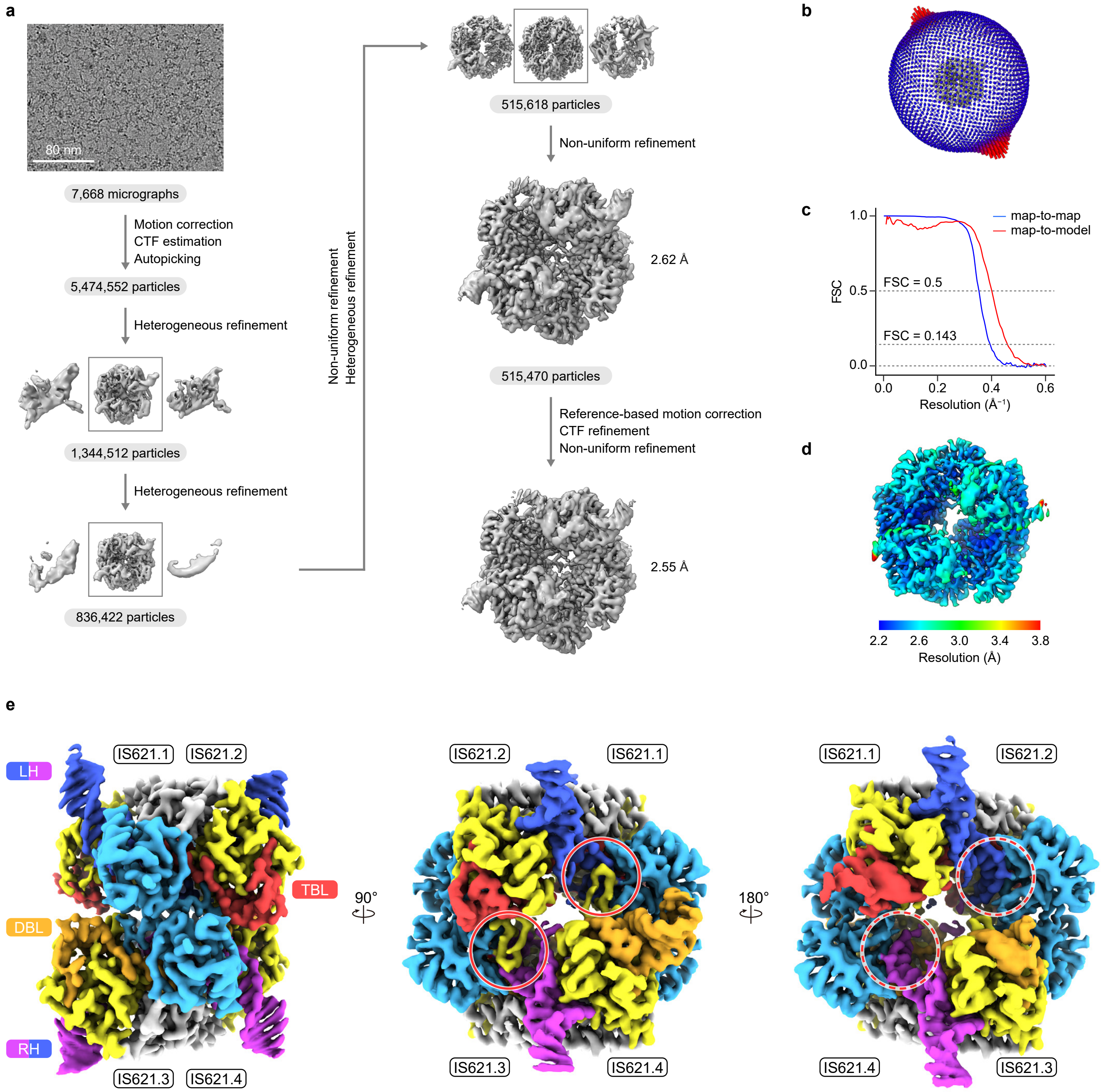

Supplementary Fig. 4 | Cryo-EM analysis of the IS621 excision complex in the post-strand exchange state.

a, Single-particle cryo-EM image processing workflow.

b, Angular distribution of particles in the final reconstruction.

c, FSC curve.

d,**e**, Cryo-EM density maps, colored according to the local resolution (**d**) and domains (**e**).

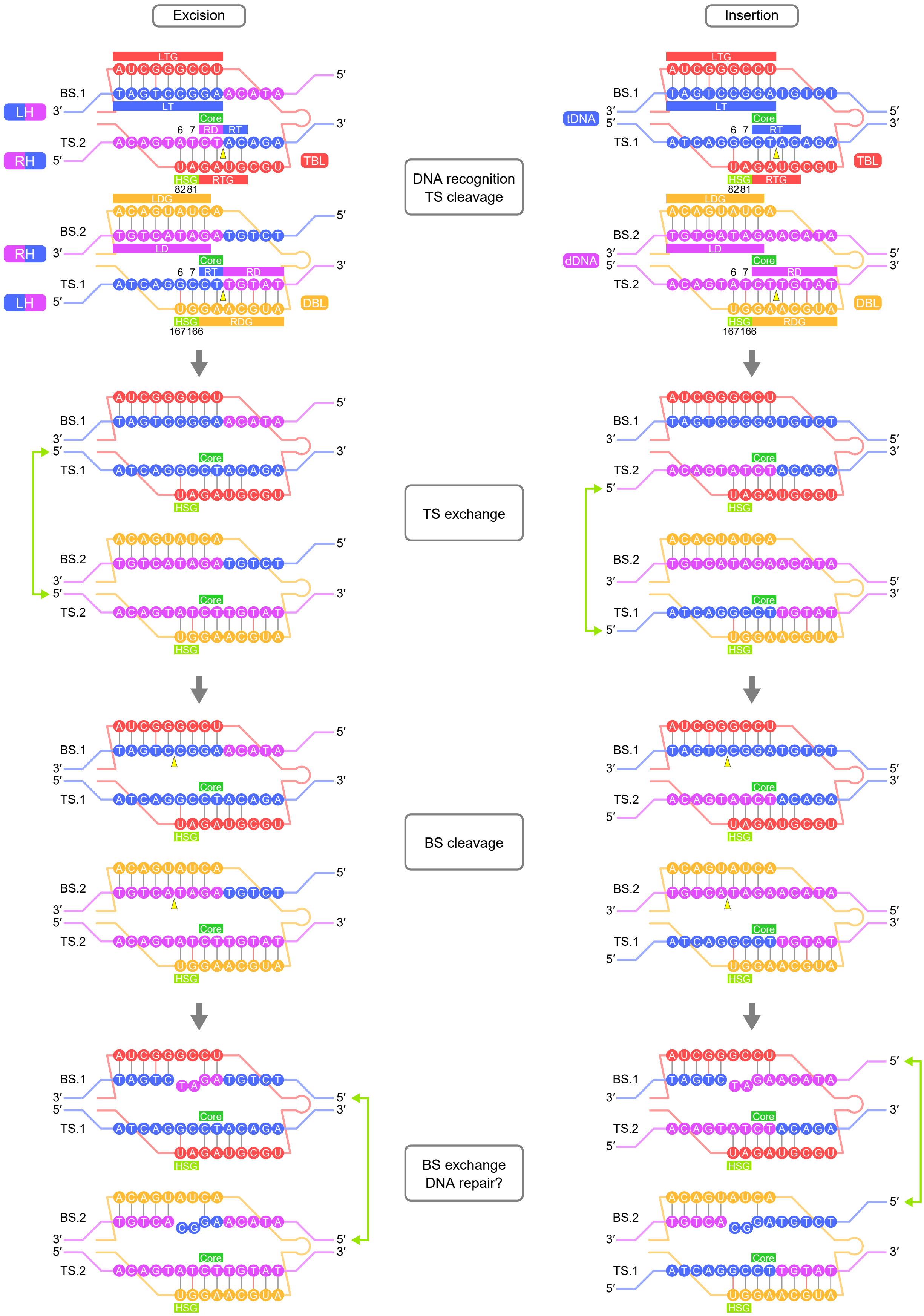

Supplementary Fig. 5 | Proposed mechanisms of IS621-mediated excision and insertion.
In both excision and insertion, the top strands of the DNA substrates base pair with the RTG and RDG in the bRNA, and are then cleaved at the RuvC–Tnp active sites (RuvC.1–Tnp.4 and RuvC.3–Tnp.2), with the catalytic S241 residues forming covalent 5′-phosphoserine intermediates. Subsequently, the top strands are exchanged and religated, followed by the cleavage of the bottom strands at other RuvC–Tnp active sites (RuvC.4–Tnp.1 and RuvC.2–Tnp.3). The bottom strands are exchanged, and mismatched nucleotides are possibly excised and repaired in *E. coli* cells, thereby completing the recombination. Non-canonical base pairs are indicated by red lines, and DNA cleavage sites are marked with yellow triangles.

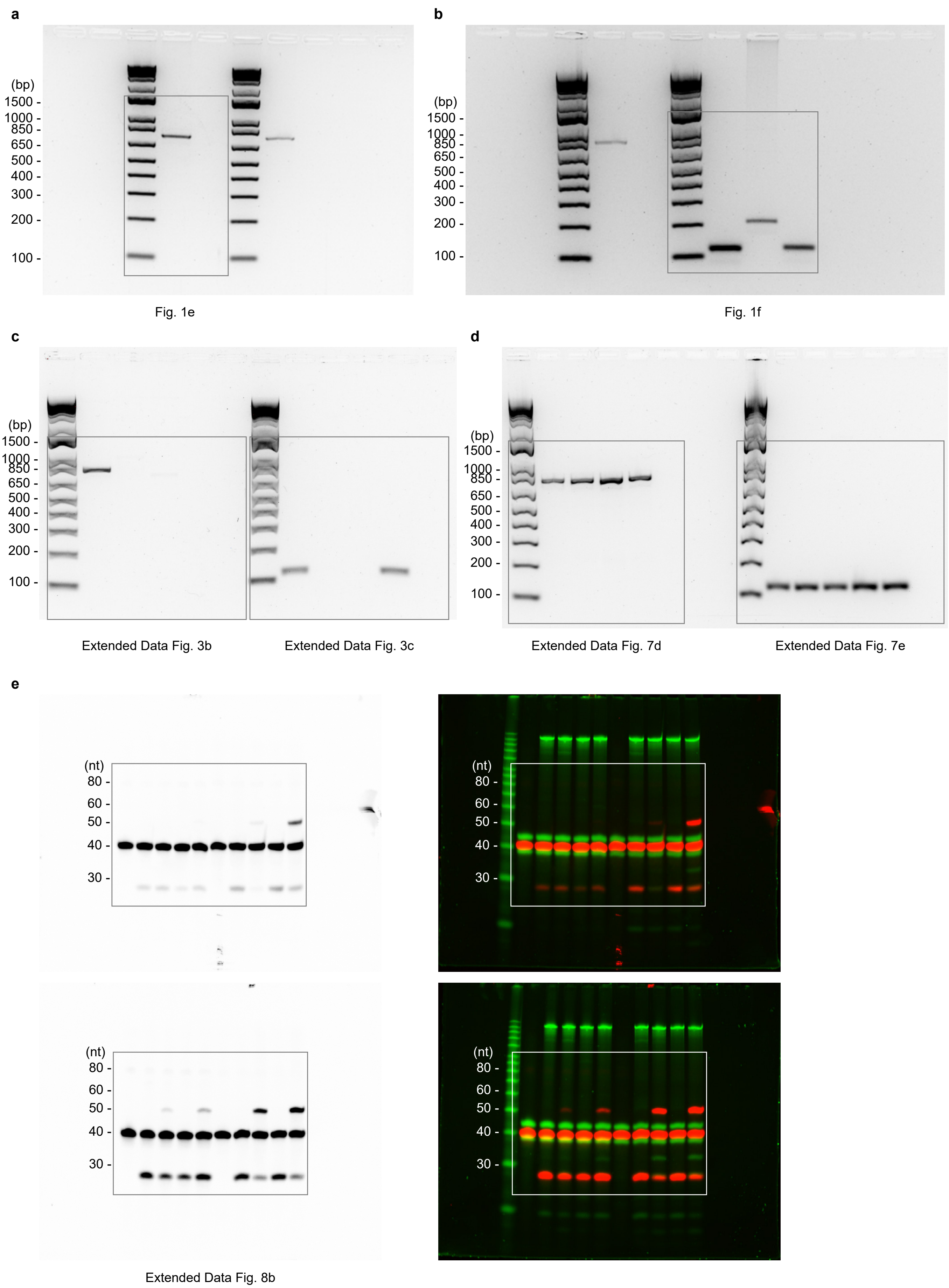

Supplementary Fig. 6 | Uncropped images of gels.

Uncropped gels used for Fig. 1e (**a**), Fig. 1f (**b**), Extended Data Figs. 3b and 3c (**c**), Extended Data Figs. 7d and 7e (**d**), and Extended Data Fig. 8b (**e**).
