# Supplementary material for: Structural mechanism governing the directionality of bridge recombination
Source: Nature. 2026 Aug 12;658(8134):269–78. doi: 10.1038/s41586-026-10903-y (PMC13626964; doi:10.1038/s41586-026-10903-y)
Supplement: Supplementary file 3 [file 41586_2026_10903_MOESM4_ESM.pdf]

nature portfolio

Peer Review File

Structural mechanism governing the directionality of bridge recombination

Corresponding Author: Professor Hiroshi Nishimasu

Any redactions in this file are there to maintain patient confidentiality, the confidentiality of unpublished data, or to remove third-party material.

This file contains all reviewer reports in order by version, followed by all author rebuttals in order by version.

Attachments originally included by the reviewers as part of their assessment can be found at the end of this file.

Version 0:

Reviewer comments:

Referee #1

(Remarks to the Author)
This is an excellent and timely study, and it can substantially push forward the bridge RNA recombinase field. Based on the team's foundational discovery and structural work on programmable bridge RNA–guided recombination (the "Bridge RNA recombinase" papers), together with the emerging applications, this manuscript provides the missing biological and mechanistic explanation for recombination directionality, especially why excision is intrinsically less favored compared with insertion. By combining in vivo evidence for basal bRNA availability, quantitative reconstitution, and cryo-EM structures, the authors present a coherent and convincing model. Overall, the work is technically solid, conceptually clear, and suited for Nature.

Minor suggestions:

1. To help readers grasp the key message more quickly, it may be helpful to add a single summary panel that succinctly connects the proposed determinants (substrate geometry/assembly/TSE) to the observed low excision efficiency.

2. For readability, I would recommend including a side-by-side table or schematic comparing excision vs insertion (e.g., overall substrate architecture, TBL/DBL engagement, potential productive vs non-productive assemblies, HSG configuration pre-/post-TSE), with a brief note on how each difference supports directionality.

3. The structural figures are highly informative; adding a few simple callouts/arrows highlighting the main directionality-related features could further improve "at-a-glance" clarity, especially for non-specialists.

Referee #2

(Remarks to the Author)
In this work, Hiraizumi and coworkers explore the molecular basis of transposon excision by the IS631 transposase and its bridge RNA (bRNA). They provide evidence that the bridge RNA and transposase are transcribed at low levels that presumably allow production of the IS631-bRNA complex that drives transposon excision. Using a collection of engineered bRNA variants, the authors then show that insertion occurs more readily than excision based on the key base-pairing interactions before and after recombination. CryoEM structures using engineered bRNAs to simulate pre-strand exchange and post-strand exchange revealed a distinct X-shaped configuration of the bound DNA in contrast to the authors' previously reported integration complex structure. These insights suggest multiple mechanisms leading to rare transposon excision followed by rapid subsequent insertion.

IS110 transposons, the source of IS631, represent recently characterized and unique recombinases that rely on the bRNA to drive transposition. While recent work including from the authors elucidated the basis of transposon insertion, far less is known about what drives excision of the transposon. The authors provide key insights from showing that the bRNA and

transposon is transcribed at low levels to elucidating highly resolved structures presumably capturing excision in action. At the same time, there are some important gaps leaving the biological relevance of their findings still unresolved, while aspects of the writing limit overall accessibility.

Major comments:

1. The authors map RNA-seq reads in and immediately upstream of the linear transposon, whether encoded in a plasmid or in the genome, as support for the conclusion that this explains IS631-bRNA production and transposon excision. However, there are gaps in this reasoning that need to be filled. First, it needs to be proven that read-through transcription underlies transposon excision, such as by inserting an upstream terminator and showing that excision is abolished. Second, any transcript would include a long 5' region compared to that expressed from the circularized transposon. This additional RNA needs to be assessed for its functionality and whether the extended end reduces transposase activity.

2. The authors rely heavily on engineered bRNAs both for measuring insertion/excision efficiencies but also when generating the cryoEM structures. This raises concerns about the biological relevance of the measurements and the structures. When probing insertion/excision frequencies, using multiple natural examples would be more compelling than solely relying on engineered bRNAs. For the structures, I recognize this was necessary to capture a pre-TS complex, although more support is needed to argue that the authors captured the excision complex rather than some other variant. If anything, wouldn't the expectation be that excision and insertion should look the same when entering a recombination reaction?

3. Based on the gained insights, can the authors achieve increased excision in vivo, such as through edits to the bRNA? This would serve as a useful final demonstration bringing biologically relevance. Similarly, can these insights be applied to understand the existing spread of bRNAs and integrated transposons?

4. I understand transposition by IS631 is complicated given the various mechanistic steps and interactions. However, the text was particularly difficult to digest given the heavy use of acronyms. The authors' prior publications on IS631 were easier to read, so it should be possible to make the text more accessible to a broad audience.

Other comments:

5. The beginning of the introduction is a slightly reworded version of the authors' 2024 Nature paper (doi: 10.1038/s41586-024-07570-2), sentence for sentence, while the first sentence of the next paragraph is exactly the same.

6. There were multiple sentences with logical incongruities. For instance, on L. 33, "these elements" should refer to the recombinase-bRNA complex, yet the elements are described as being excised. Separately, L. 66-67 describes how transposition into a new site results in the formation of LT-RD and RT junctions, although the junctions should be resolved for transposition should be complete. Finally, L. 158-159 describes an inverse relationship between sequences and a quantitative measure, although something needs to be defined about those sequences; in this case, the authors may be referring to the extent of HSG-DNA base pairing. I recommend closely rereading the manuscript for any additional instances.

7. L. 47-48: I didn't encounter any text describing how the authors' insights lend to better engineering IS163 and bRNAs for genome editing.

8. L. 88: Be more concrete on what the prior studies showed regarding excision.

9. Fig. 1d: Where does transcription start in each of those loci? Showing mapped reads extending upstream would provide better context.

10. Fig. 1e,f: Can circular and post-excision products be detected for the plasmid construct containing the linear transposon? This would provide further support for excision.

11. L. 121-149: The conclusion that the IS163-bRNA complex mediates excision less efficiently than insertion could be achieved with a far smaller set of engineered bRNAs (or even none of the engineered bRNAs).

12. L. 129-130: Use of "significant" should come with statistical analyses. Also, in ED Fig. 4b, only RTG extensions provided a consistent enhancement.

13. L. 131-132: Based on the presented data in ED Fig. 5a, the TBL was sufficient.

Referee #3

(Remarks to the Author)
I co-reviewed this manuscript with one of the reviewers who provided the listed reports.

Referee #4

(Remarks to the Author)
I co-reviewed this manuscript with one of the reviewers who provided the listed reports.

Referee #5

(Remarks to the Author)
The manuscript by Hiraizumi et al. ,"Structural mechanism governing the directionality of IS110 bridge recombination", focuses on bridge recombinases from the IS110 family of transposons. To date, IS110 bridge RNA (bRNA)-guided recombination has been primarily characterized in the context of insertion reactions mediated by recombinase-bRNA complexes, while the mechanism underlying excision and circular intermediate formation has remained unclear. This study directly addresses the missing gap.

More specifically, through a combination of biochemical and structural work, the authors demonstrate that the bRNA is expressed from IS621 genomic loci and provide the evidence that the IS621 recombinase-bRNA complex is capable of mediating excision, albeit with lower efficiency than insertion. By solving structures of the excision complex bound to DNA substrates, the authors reveal a distinct mode of DNA recognition compared to the insertion complex. The identification of X-shaped excision substrates engaging both bRNA loops offers a structural explanation for the observed bias against excision and suggests the unifying mechanism on how bRNA-DNA base pairing modulates both reaction directions.

The study is technically sound and the conclusions are well supported by the experimental data. Additionally, it is a very timely study, and the results may be of interest not only to researchers studying transposons and mobile genetic elements, but also to those working in the genome editing field, where IS110-derived bRNA-guided recombinases have emerged as a novel tool for genome manipulation. Overall, I have only minor comments/suggestions.

Minor comments:

The manuscript is overly focused on structural details, both in the text and in the number of figure panels that are not essential to the main message or conclusions of the study. The authors are advised to reconsider which parts and details are essential to include in order to better emphasize the study key findings.

P3. Line 59-61, "Unlike typical IS elements, IS110 family members <…> possess a unique architecture comprising a left end (LE), a recombinase gene, and a right end (RE).": Other insertion sequences (e.g., IS200) have a similar structure; therefore it is unclear what specific architectural features the authors consider to be unique to the IS110 family.

P5. Line 124, "…such as RTG and RDG extension (positions 74–76 and 161)": Please clarify whether position 11 should also be included, as suggested by Ext. Data Fig. 3.

P5. Line 126-127, "…and 86-bp DNA substrates (an LH DNA with LT–RD and an RH DNA with LD–RT for excision, and a tDNA with LT–RT and a dDNA with LD–RD for insertion)": The lengths of the excision substrates shown in Ext. Data Fig. 4 appear to differ from 86 bp.

P5. Line 136-137. The authors state that "Intriguingly, our bRNA modifications that increased the insertion efficiencies universally decreased the excision efficiencies (Extended Data Fig. 4b)". The reduction in excision efficiency appears to be modest and may fall within the margin of experimental error. The authors should clarify/explain this point.

PP9-10. Lines 306-308, "During insertion, the IS621–TBL–tDNA dimer and the IS621–DBL–dDNA dimer assemble into a functional tetramer…": The authors should clarify the stoichiometry of the individual components, as the use of the term "dimer" in this context may be misleading.

RNA sequencing data should be deposited in the public database.

Version 1:

Reviewer comments:

Referee #2

(Remarks to the Author)
The authors have reasonably addressed all raised comments with a combination of new experimental results reinforcing their original conclusions as well as changes to the text to improve clarity and accessibility. I have no more comments, and I commend the authors on their work uncovering the basis of transposon excision and fully engaging in the peer-review process.

Referee #3

(Remarks to the Author)
I co-reviewed this manuscript with one of the reviewers who provided the listed reports.

Referee #4

(Remarks to the Author)

I co-reviewed this manuscript with one of the reviewers who provided the listed reports.

Referee #5

(Remarks to the Author)
All my minor concerns have been addressed by the authors.

Open Access This Peer Review File is licensed under a Creative Commons Attribution 4.0 International License, which permits use, sharing, adaptation, distribution and reproduction in any medium or format, as long as you give appropriate credit to the original author(s) and the source, provide a link to the Creative Commons license, and indicate if changes were made.
In cases where reviewers are anonymous, credit should be given to 'Anonymous Referee' and the source.
The images or other third party material in this Peer Review File are included in the article’s Creative Commons license, unless indicated otherwise in a credit line to the material. If material is not included in the article’s Creative Commons license and your intended use is not permitted by statutory regulation or exceeds the permitted use, you will need to obtain permission directly from the copyright holder.
To view a copy of this license, visit https://creativecommons.org/licenses/by/4.0/

We greatly appreciate the reviewers' suggestions and concerns, which have significantly improved the quality of our work. According to the reviewers' comments, we have revised the manuscript and hope that it is now acceptable for publication in *Nature*.

The major changes and revisions are as follows:

In response to Reviewer 2, we added new data demonstrating that the deletion of the bRNA from the left end of the element, as well as the insertion of a potent transcriptional terminator upstream of the left end, abolished excision in *E. coli* (new Extended Data Fig. 3) We further confirmed that bRNAs with extended 5′ and/or 3′ ends, as would be produced by read-through transcription, support both excision and insertion *in vitro* (new Extended Data Fig. 6b). We also demonstrated that the excision efficiency could be substantially increased in *E. coli*, using an engineered bRNA with a post-TSE configuration, providing *in vivo* validation of our mechanistic findings (new Extended Data Fig. 7b–e). Furthermore, we have improved the readability of the manuscript through substantial textual revisions addressing concerns about the accessibility for a broader audience.

Referee #1:

This is an excellent and timely study, and it can substantially push forward the bridge RNA recombinase field. Based on the team's foundational discovery and structural work on programmable bridge RNA–guided recombination (the "Bridge RNA recombinase" papers), together with the emerging applications, this manuscript provides the missing biological and mechanistic explanation for recombination directionality, especially why excision is intrinsically less favored compared with insertion. By combining in vivo evidence for basal bRNA availability, quantitative reconstitution, and cryo-EM structures, the authors present a coherent and convincing model. Overall, the work is technically solid, conceptually clear, and suited for Nature.

Thank you for your very positive assessment of our work!

Minor suggestions:

1. To help readers grasp the key message more quickly, it may be helpful to add a single summary panel that succinctly connects the proposed determinants (substrate geometry/assembly/TSE) to the observed low excision efficiency.

Thank you for the suggestion. We have revised Fig. 5 to highlight the key determinants of efficiency during both excision and insertion processes.

2. For readability, I would recommend including a side-by-side table or schematic comparing excision vs insertion (e.g., overall substrate architecture, TBL/DBL engagement, potential productive vs non-productive assemblies, HSG configuration pre-/post-TSE), with a brief note on how each difference supports directionality.

Thank you for the suggestion. We have added information, such as overall substrate architecture and HSG–DNA base pairing before and after TS exchange, to clarify the differences between the excision and insertion processes.

3. The structural figures are highly informative; adding a few simple callouts/arrows highlighting the main directionality-related features could further improve "at-a-glance" clarity, especially for

non-specialists.

Thank you for the comment. To enhance the clarity of structural figures, we have modified several figures, including Figs. 2b, 2e, 2f, 3b–d, 4c, 4d, 5b, and Extended Data Fig. 11, to highlight the HSG and core nucleotides by differentiating the nucleobase models and/or their coloring (with the HSG depicted in yellow-green and the core in green).

Referee #2:

In this work, Hiraizumi and coworkers explore the molecular basis of transposon excision by the IS631 transposase and its bridge RNA (bRNA). They provide evidence that the bridge RNA and transposase are transcribed at low levels that presumably allow production of the IS631-bRNA complex that drives transposon excision. Using a collection of engineered bRNA variants, the authors then show that insertion occurs more readily than excision based on the key base-pairing interactions before and after recombination. CryoEM structures using engineered bRNAs to simulate pre-strand exchange and post-strand exchange revealed a distinct X-shaped configuration of the bound DNA in contrast to the authors' previously reported integration complex structure. These insights suggest multiple mechanisms leading to rare transposon excision followed by rapid subsequent insertion.

IS110 transposons, the source of IS631, represent recently characterized and unique recombinases that rely on the bRNA to drive transposition. While recent work including from the authors elucidated the basis of transposon insertion, far less is known about what drives excision of the transposon. The authors provide key insights from showing that the bRNA and transposon is transcribed at low levels to elucidating highly resolved structures presumably capturing excision in action. At the same time, there are some important gaps leaving the biological relevance of their findings still unresolved, while aspects of the writing limit overall accessibility.

Thank you for your positive assessment of our work.

Major comments:

1. The authors map RNA-seq reads in and immediately upstream of the linear transposon, whether encoded in a plasmid or in the genome, as support for the conclusion that this explains IS631-bRNA production and transposon excision. However, there are gaps in this reasoning that need to be filled. First, it needs to be proven that read-through transcription underlies transposon excision, such as by inserting an upstream terminator and showing that excision is abolished. Second, any transcript would include a long 5' region compared to that expressed from the circularized transposon. This additional RNA needs to be assessed for its functionality and whether the extended end reduces transposase activity.

We thank the reviewer for raising these important points, which we have fully addressed. To demonstrate that read-through transcription underlies excision, we inserted a BBa_B1006 high-efficiency transcriptional terminator upstream of the left end of the element on a plasmid and confirmed that excision was abolished in *E. coli* BL21(DE3) (which lacks endogenous IS621 elements), as assessed by PCR targeting both the post-excision junction and the expected circular intermediate (new Extended Data Fig. 3). Deletion of the bRNA from the LE yielded identical results. We next assessed whether extended bRNA transcripts, as would arise from read-through transcription, could support recombination *in vitro*. Using transcripts up to 775 nucleotides in length, with the 5′ end, the 3′

end, or both ends extended based on a putative TSS mapped to an upstream CDS (new Fig. 1d and Extended Data Fig. 2c), we found that the extended bRNAs are compatible with both excision and insertion (new Extended Data Fig. 6b). When both ends were extended to mimic read-through from the TSS of an upstream gene into the IS621 recombinase CDS, the excision and insertion efficiencies were reduced approximately 2-fold relative to the minimal bRNA.

2. The authors rely heavily on engineered bRNAs both for measuring insertion/excision efficiencies but also when generating the cryoEM structures. This raises concerns about the biological relevance of the measurements and the structures. When probing insertion/excision frequencies, using multiple natural examples would be more compelling than solely relying on engineered bRNAs. For the structures, I recognize this was necessary to capture a pre-TS complex, although more support is needed to argue that the authors captured the excision complex rather than some other variant. If anything, wouldn't the expectation be that excision and insertion should look the same when entering a recombination reaction?

We thank the reviewer for this important point. We respectfully note that *in vitro* experiments examining the role of handshake guides in recombination directionality have been repeated using naturally occurring bRNAs without additional scaffold engineering, and the patterns and conclusions are consistent across both contexts. For example, Extended Data Fig. 7a (effects of HSG base pairing in the engineered bRNA; Extended Data Fig. 5c in the original manuscript) largely mirrors Fig. 1i (effects of HSG base pairing in the wild-type bRNA); the inverse relationship between HSG configuration and excision versus insertion efficiency is preserved across both the wild-type and engineered bRNA scaffolds, with the engineered scaffold resulting in higher absolute efficiencies and a cleaner background for dissecting the mechanistic contribution of HSG base pairing. We chose to focus on the engineered bRNA in the main figures because it allows us to isolate the specific contribution of HSG base pairing to recombination directionality, independent of other sequence features such as the non-canonical base pair between bRNA (G53) and tDNA (T11*) present in the wild-type scaffold, which also influences the efficiency (Extended Data Fig. 5b; original Extended Data Fig. 4b). Importantly, the wild-type bRNA data confirm that the same directional trends hold in the natural sequence context, validating the conclusions drawn from the engineered system (Extended Data Fig. 7a; original Extended Data Fig. 5c). We believe this focus is warranted not only for mechanistic clarity but also because engineered constructs provide the relevant context for genome engineering applications, where only these optimized variants can achieve efficient recombination. Similarly, the use of split bRNA for structural studies was essential to capture the desired functional states.

With respect to the structures and whether we captured the excision complex, rather than some other variant, we direct the reviewer to Fig. 5b: the excision substrates adopt an X-shaped configuration as a direct consequence of simultaneous base pairing between each bRNA loop and both the LH and RH substrates. This geometry is not an arbitrary conformational variant, but is mechanistically required: the LH and RH each contain both LT/RD and LD/RT sequences, respectively, which must engage both the TBL and DBL to position the CT cores at the composite RuvC–Tnp active sites. A U-shaped insertion-like configuration is geometrically incompatible with this dual-loop engagement and would not permit the observed RNA–DNA base pairing.

Regarding the reviewer's expectation that excision and insertion should look the same at the start of a recombination reaction, this symmetry is indeed present in our data, but it exists between the insertion post-strand exchange state and the excision pre-strand exchange state, not between the pre-strand exchange states of both reactions (Fig. 5b). This relationship is not incidental; it is a central mechanistic

finding of the paper. The thermodynamically unfavorable transition from the X-shaped excision substrates to a strand-exchanged configuration, contrasted with the favorable relaxation from the bent U-shaped insertion substrates, directly explains the lower efficiency of excision. This is further supported by Extended Data Figure 8b (original Extended Data Figure 6b), which shows comparable levels of top-strand cleavage intermediates in both reactions but substantially less recombination product in excision, demonstrating that the efficiency difference arises specifically at the strand-exchange step, rather than at the cleavage step.

3. Based on the gained insights, can the authors achieve increased excision in vivo, such as through edits to the bRNA? This would serve as a useful final demonstration bringing biologically relevance. Similarly, can these insights be applied to understand the existing spread of bRNAs and integrated transposons?

We thank the reviewer for this suggestion. We have now added data (new Extended Data Fig. 7b–e) demonstrating that the engineered bRNA with a post-TSE configuration (for excision), which yielded the greatest increase in excision efficiency in our *in vitro* studies, also increases the excision efficiency approximately 13,000-fold relative to the wild-type bRNA, which has a pre-TSE configuration (for excision), when the element is encoded on a plasmid in *E. coli* BL21(DE3) cells lacking endogenous IS621 elements. This provides direct *in vivo* validation of our mechanistic findings, confirming that the engineering principles derived from our biochemical and structural analyses, including the HSG configuration, the RDG/RTG extensions, and the G53A modification, translate to a biological setting.

Regarding the spread of naturally occurring bRNAs and integrated transposons, our findings offer a mechanistic framework for understanding the evolutionary tuning of IS110 elements. As demonstrated in this study, the HSG configuration is a key determinant of recombination directionality; the optimal configuration for any given reaction promotes a net gain in RNA–DNA base pairs after top-strand exchange. Because the DNA substrates at positions 6–7 differ between excision and insertion, an HSG configuration that is post-TSE for one reaction is necessarily pre-TSE for the other. As noted in the manuscript, the wild-type HSG base-pairing pattern is highly conserved among IS110 family members and corresponds to a post-TSE configuration for insertion but a pre-TSE configuration for excision, consistent with evolutionary selection to favor insertion and suppress excision, thereby promoting stable transposon maintenance in prokaryotic genomes. Conversely, elements with HSG sequences that adopt a post-TSE configuration for excision would be expected to excise more readily, but at the cost of reduced insertion efficiency—a trade-off that would disfavor long-term genomic persistence. We hope that the mechanistic framework established here will motivate future comparative studies across IS110 family members to test these predictions directly.

4. I understand transposition by IS631 is complicated given the various mechanistic steps and interactions. However, the text was particularly difficult to digest given the heavy use of acronyms. The authors' prior publications on IS631 were easier to read, so it should be possible to make the text more accessible to a broad audience.

We appreciate this feedback and have revised the manuscript to improve readability throughout. We note that most acronyms used in this study (TBL, DBL, LT, RT, LD, RD, LTG, RTG, LDG, RDG, and HSG) were established in our prior publications and are necessary to describe the mechanistic features of the bridge recombination system. The only new terms introduced in this manuscript are LH (left half) and RH (right half), which describe the excision substrate junctions, and the pre- and post-TSE (top-strand exchange) concepts, which describe the two HSG configurations relative to top-strand

exchange. To improve accessibility, we have ensured that all acronyms are clearly defined at first use, reintroduced at key points in the text, and spelled out in contexts where they appear infrequently. We have also restructured several passages to improve the narrative flow for a broad audience.

Other comments:

5. The beginning of the introduction is a slightly reworded version of the authors' 2024 Nature paper (doi: 10.1038/s41586-024-07570-2), sentence for sentence, while the first sentence of the next paragraph is exactly the same.

We thank the reviewer for this observation. We have completely rewritten the introduction to provide a distinct framing that contextualizes the excision mechanism as the central focus of this study, rather than recapitulating the background presented in our 2024 Nature paper.

6. There were multiple sentences with logical incongruities. For instance, on L. 33, "these elements" should refer to the recombinase-bRNA complex, yet the elements are described as being excised. Separately, L. 66-67 describes how transposition into a new site results in the formation of LT-RD and RT junctions, although the junctions should be resolved for transposition should be complete. Finally, L. 158-159 describes an inverse relationship between sequences and a quantitative measure, although something needs to be defined about those sequences; in this case, the authors may be referring to the extent of HSG-DNA base pairing. I recommend closely rereading the manuscript for any additional instances.

We thank the reviewer for their careful reading. We have revised the manuscript throughout to address these and other instances of imprecise language. Regarding the specific points raised: (1) We have revised the abstract to clearly distinguish between IS621 elements (which are excised) and the recombinase–bRNA complex (which mediates the reaction). (2) We note that "junction" is standard terminology in the recombination field for describing the boundary between two joined DNA sequences at an insertion site. LH and RH refer to the LT–RD and LD–RT junctions that define the boundaries of the inserted element, rather than unresolved recombination intermediates. We have ensured this is clearly defined in the revised text. (3) We have revised the description to specify "the extent of HSG–DNA base pairing" rather than "the HSG sequences," to make it explicit that it is the degree of base pairing, not the sequences per se, that correlates with efficiency. We have carefully reread the manuscript to identify and correct additional instances of imprecise language.

7. L. 47-48: I didn't encounter any text describing how the authors' insights lend to better engineering IS163 and bRNAs for genome editing.

We thank the reviewer for highlighting this gap. We have added text to the Discussion clarifying how the mechanistic insights reported here translate to practical bRNA engineering for genome editing applications. Specifically, we note that several of the modifications characterized in this study, including the split bRNA system and RTG/RDG extensions, have already been applied to enhance recombination efficiency in human cells (Perry *et al.*, *Science*, 2025), providing a concrete demonstration of how these findings facilitate the optimal design of bridge recombinase tools for programmable genome editing.

8. L. 88: Be more concrete on what the prior studies showed regarding excision.

We thank the reviewer for this suggestion. We have expanded on the previously cited references (Siddiquee *et al.*, *Nat. Commun.*, 2024; Parés-Guillén *et al.*, *Nucleic Acids Res.*, 2025) to concretely describe what each study demonstrated regarding excision in IS110 family members, providing a clearer context for the open mechanistic questions that our work addresses.

9. Fig. 1d: Where does transcription start in each of those loci? Showing mapped reads extending upstream would provide better context.

To better illustrate the genomic context of each IS621 locus, including candidate upstream transcription start sites that could give rise to read-through transcripts encoding the initial bRNA required for excision, we have revised Fig. 1d and added the new Extended Data Fig. 2c showing the upstream genomic context and predicted TSS for each site. The TSS upstream of the CDS proximal to IS621-1 was used as the starting point for the 5′ extension of the bRNA in our *in vitro* experiments evaluating whether read-through transcription-derived bRNA could support recombination (new Extended Data Fig. 6b).

10. Fig. 1e,f: Can circular and post-excision products be detected for the plasmid construct containing the linear transposon? This would provide further support for excision.

We thank the reviewer for this question. Yes, both the post-excision junction and the circular intermediate can be detected by PCR for the plasmid construct encoding the linear IS621 element, both in the absence of an immediately proximal upstream promoter and when driven by an upstream T7 promoter, as shown in the new Extended Data Fig. 7d,e. The RNA sequencing data demonstrate low-level transcription across the linear element even in the absence of a proximal promoter, likely arising from read-through transcription originating from distal promoters elsewhere on the plasmid, such as the KanR cassette. Together, these data are consistent with the model that read-through transcription is sufficient to generate the bRNA required to initiate excision from the linear form of the element, even when contained within a plasmid.

11. L. 121-149: The conclusion that the IS163-bRNA complex mediates excision less efficiently than insertion could be achieved with a far smaller set of engineered bRNAs (or even none of the engineered bRNAs).

We acknowledge the reviewer's point that the core conclusion that the IS621–bRNA complex mediates excision less efficiently than insertion is already evident from the wild-type bRNA data alone, where the excision efficiency (0.12%) is approximately 50-fold lower than the insertion efficiency (6.2%) (Extended Data Fig. 5b; original Extended Data Fig. 4b). Furthermore, the role of HSG base pairing in modulating this directionality was demonstrated using the wild-type bRNA scaffold in Extended Data Fig. 7a (original Extended Data Fig. 5c), without requiring any engineered variants. However, we believe the full engineered bRNA panel presented in Extended Data Fig. 5b (original Extended Data Fig. 4b) provides important additional insights that go beyond these conclusions. Specifically, the systematic characterization of individual bRNA modifications reveals that changes which enhance insertion universally suppress excision and vice versa, an inverse relationship that provides key mechanistic insight into how the system balances recombination directionality and that directly motivated our structural studies and *in vivo* engineering experiments. These engineered constructs, including split bRNA, are also the relevant context for genome engineering applications in human cells, as demonstrated in our recent study (Perry *et al.*, *Science*, 2025), further broadening the relevance of this work beyond transposon biology. We have nevertheless considered the reviewer's comment

seriously and have made efforts to improve the clarity and accessibility of this section in the revised manuscript.

12. L. 129-130: Use of “significant” should come with statistical analyses. Also, in ED Fig. 4b, only RTG extensions provided a consistent enhancement.

We have revised the text to replace “significant” with “substantial” and updated the description to more accurately reflect the data. Specifically, the RTG extension alone provided a substantial enhancement in insertion efficiency (from 6.2% to 57%), while the RDG extension alone produced a less consistent enhancement (6.2% to 6.4%). However, combining both the RTG and RDG extensions yielded a clear additive enhancement (6.2% to 77%), and we have revised the text to reflect this nuanced observation.

The revised text now reads: “Consistent with our previous findings, we observed substantial enhancements in insertion efficiency with the RTG extension, with further additive improvement in combination with the RDG extension (Extended Data Fig. 5b).”

13. L. 131-132: Based on the presented data in ED Fig. 5a, the TBL was sufficient.

We clarify that the text states that “efficient” insertion requires both the TBL and DBL, a distinction we may not have stated clearly enough in the original text. The data in Extended Data Fig. 6a (original Extended Data Fig. 5a) support this; the insertion efficiency with TBL alone is 0.003%, and that with the engineered TBL alone is 0.02%, values that are close to the no-bRNA background of 0.001% and therefore likely within the noise of the qPCR assay for insertion. In contrast, the combination of TBL and DBL yields 5% efficiency, rising to 72% with both engineered TBL and engineered DBL. While the TBL alone may produce a detectable signal above background, the dramatic enhancement upon the inclusion of the DBL demonstrates that both loops are required for efficient recombination. We believe the text accurately reflects this distinction and no revision is necessary on this point.

Referee #3:
I co-reviewed this manuscript with one of the reviewers who provided the listed reports.

Referee #4:
I co-reviewed this manuscript with one of the reviewers who provided the listed reports.

Referee #5:

The manuscript by Hiraizumi et al. ,“Structural mechanism governing the directionality of IS110 bridge recombination”, focuses on bridge recombinases from the IS110 family of transposons. To date, IS110 bridge RNA (bRNA)-guided recombination has been primarily characterized in the context of insertion reactions mediated by recombinase-bRNA complexes, while the mechanism underlying excision and circular intermediate formation has remained unclear. This study directly addresses the missing gap.

More specifically, through a combination of biochemical and structural work, the authors demonstrate that the bRNA is expressed from IS621 genomic loci and provide the evidence that the IS621 recombinase-bRNA complex is capable of mediating excision, albeit with lower efficiency than insertion. By solving structures of the excision complex bound to DNA substrates, the authors reveal a distinct mode of DNA recognition compared to the insertion complex. The identification of X-shaped excision substrates engaging both bRNA loops offers a structural explanation for the observed bias

against excision and suggests the unifying mechanism on how bRNA-DNA base pairing modulates both reaction directions.

The study is technically sound and the conclusions are well supported by the experimental data. Additionally, it is a very timely study, and the results may be of interest not only to researchers studying transposons and mobile genetic elements, but also to those working in the genome editing field, where IS110-derived bRNA-guided recombinases have emerged as a novel tool for genome manipulation. Overall, I have only minor comments/suggestions.

Thank you for your positive assessment of our work.

Minor comments:

The manuscript is overly focused on structural details, both in the text and in the number of figures that are not essential to the main message or conclusions of the study. The authors are advised to reconsider which parts and details are essential to include in order to better emphasize the study key findings.

We appreciate the reviewer's comment and agree that the original manuscript included non-essential structural details. Accordingly, we have removed redundant descriptions about the excision complex structure (*e.g.*, overall structure, bRNA recognition, and active-site architecture) that were already described in our previous paper (Hiraizumi *et al.*, *Nature*, 2024), to emphasize the key findings of this study. We also removed the *in vitro* recombination data on excision programmability (previous Extended Data Fig. 5b), as they are not essential to the main message. Instead, in response to Referee 2's suggestions, we added new functional data that strengthened our conclusions (Extended Data Figs. 3, 6b, and 7b–e). To enhance the clarity of the structural figures, we have modified several figures, including Figs. 2b, 2e, 2f, 3b–d, 4c, 4d, 5b, and Extended Data Fig. 11, to highlight the HSG and core nucleotides. Additionally, we have revised Fig. 5 to clarify the differences between the excision and insertion processes.

P3. Line 59-61, "Unlike typical IS elements, IS110 family members <…> possess a unique architecture comprising a left end (LE), a recombinase gene, and a right end (RE).": Other insertion sequences (e.g., IS200) have a similar structure; therefore it is unclear what specific architectural features the authors consider to be unique to the IS110 family.

We thank the reviewer for this point. In the revised manuscript, we have rewritten this sentence to remove the claim of unique architecture. The revised text now reads: "Unlike typical transposable elements, IS110 elements [...] comprise a left end (LE), a recombinase gene, and a right end (RE), with conserved CT (cytidine-thymidine) core dinucleotide sequences at both boundaries." This framing emphasizes the combination of architectural and sequence features that distinguish IS110 elements, rather than implying that the LE–recombinase–RE organization is itself without precedent.

P5. Line 124, "…such as RTG and RDG extension (positions 74–76 and 161)": Please clarify whether position 11 should also be included, as suggested by Ext. Data Fig. 3.

We thank the reviewer for this observation. Position 11 corresponds to the G53A substitution. As shown in Extended Data Fig. 5b (original Extended Data Fig. 4b), G53A combined with RTG/RDG extensions yields 63% insertion efficiency, which is lower than RTG/RDG extensions alone (77%). Because G53A does not enhance insertion in this context, we highlighted only RTG/RDG extensions in

the main text. Furthermore, this section has been revised for clarity, and the full set of modifications is detailed in Extended Data Fig. 4 (original Extended Data Fig. 3).

P5. Line 126-127, "…and 86-bp DNA substrates (an LH DNA with LT–RD and an RH DNA with LD–RT for excision, and a tDNA with LT–RT and a dDNA with LD–RD for insertion)": The lengths of the excision substrates shown in Ext. Data Fig. 4 appear to differ from 86 bp.

We thank the reviewer for catching this error. The excision substrates are not 86 bp. We have corrected the text to specify the actual lengths: "We used the purified IS621 recombinase and the 177-nt bRNA with DNA substrates for excision (51-bp LH and 121-bp RH) or insertion (86-bp tDNA and dDNA)."

P5. Line 136-137. The authors state that "Intriguingly, our bRNA modifications that increased the insertion efficiencies universally decreased the excision efficiencies (Extended Data Fig. 4b)". The reduction in excision efficiency appears to be modest and may fall within the margin of experimental error. The authors should clarify/explain this point.

We thank the reviewer for raising this point. We note that all excision and insertion efficiency values reported in Extended Data Fig. 5b (original Extended Data Fig. 4b) have been corrected by subtracting the no-bRNA control background signal, and that the measured excision efficiencies for each condition are above the background of the assay. While some individual reductions in excision efficiency are modest in absolute terms, the trend is consistent across all modifications tested, with no exceptions. Furthermore, the combined effect is substantial; for instance, the fully modified bRNA (G53A substitution and RTG/RDG extensions) reduced excision approximately 4-fold relative to WT (0.03% for the engineered bRNA vs. 0.12% for the WT bRNA), while leading to a 10-fold increase in insertion (63% for the engineered bRNA vs. 6.2% for the WT bRNA). We have revised the wording to "every bRNA modification that increased the insertion efficiency caused a corresponding decrease in the excision efficiency" to more accurately describe this observation.

PP9-10. Lines 306-308, "During insertion, the IS621–TBL–tDNA dimer and the IS621–DBL–dDNA dimer assemble into a functional tetramer…": The authors should clarify the stoichiometry of the individual components, as the use of the term "dimer" in this context may be misleading.

We thank the reviewer for this point. We have revised this passage to clarify that "dimer" refers to a pair of IS621 recombinase protomers. Specifically, we replaced the hyphenated nomenclature (*e.g.*, "IS621–TBL–tDNA dimer") with "IS621 recombinase dimers bound to TBL/tDNA and DBL/dDNA," making the recombinase stoichiometry explicit at each step of the assembly description.

RNA sequencing data should be deposited in the public database.

We have submitted the RNA sequencing data to the NCBI Gene Expression Omnibus (GEO), with the accession number GSE328474.
